# microRNA-27a-3p delivered by extracellular vesicles from glioblastoma cells induces M2 macrophage polarization via the *EZH1/KDM3A/CTGF* axis

**DOI:** 10.1038/s41420-022-01035-z

**Published:** 2022-05-14

**Authors:** Guifang Zhao, Hongquan Yu, Lijuan Ding, Weiyao Wang, Huan Wang, Yao Hu, Lingsha Qin, Guangce Deng, Buqing Xie, Guofeng Li, Ling Qi

**Affiliations:** 1 The Sixth Affiliated Hospital of Guangzhou Medical University, Qingyuan People’s Hospital, Qingyuan, 511518 China; 2 Jilin Medical University, Jilin, 132013 China; 3 Department of Oncological Neurosurgery, the First Hospital of Jilin University, Changchun, 130021 China

**Keywords:** Cancer epigenetics, Cancer metabolism

## Abstract

Glioblastoma (GBM) cell-derived extracellular vesicles (EVs) have been demonstrated to modulate tumor microenvironment. In the present study, we attempted to discuss the role of hsa-microRNA-27a-3p (miR-27a-3p) delivered by GBM-EVs in M2 macrophage polarization. The isolated GBM-EVs were co-cultured with macrophages. After co-culture under normoxia/hypoxia, the effect of EV-derived hsa-miR-27a-3p on GBM cell biological processes was analyzed. Additionally, the target genes of hsa-miR-27a-3p were predicted. Moreover, the binding of enhancer of *zeste homologue 1* (*EZH1*) to *lysine-specific demethylase 3A* (*KDM3A*) promoter region and the interaction between *KDM3A* and *connective tissue growth factor* (*CTGF*) were analyzed. GBM mouse models were established to verify the functions of EV-derived hsa-miR-27a-3p in vivo. We found increased hsa-miR-27a-3p in GBM tissues as well as GBM-EVs, which induced M2 polarization, thus promoting proliferative, migrative and invasive potentials of GBM cells. hsa-miR-27a-3p targeted *EZH1* and promoted *KDM3A* expression to elevate the *CTGF* expression. GBM-EV-delivered hsa-miR-27a-3p promoted the *KDM3A*-upregulated *CTGF* by downregulating *EZH1*, thereby promoting M2 macrophage polarization and development of GBM in vivo. We demonstrated that EV-derived hsa-miR-27a-3p may promote M2 macrophage polarization to induce GBM.

## Introduction

Glioblastoma (GBM) is the most lethal type of glioma with aggressive brain tumors and often occurs in individuals older than 65 years [1, 2]. Moreover, the median survival of patients with GBM is shorter than two years [3], highly suggestive of the urgent need to develop novel treatment modalities. Tumor-associated M2 macrophages are reported to engage in tumor development [4]. Hypoxia and M2-like macrophages are correlated with poor prognosis of patients with GBM [5]. Therefore, to investigate the M2 macrophage-modulated tumor microenvironment of GBM may represent a prognostic biomarker for GBM. It is reported that the extracellular vesicles (EVs) of GBM (GBM-EVs) carry functional genomic and proteomic cargoes and affect surrounding and distant recipient cells, which enables EVs to emerge as crucial mediators of tumor microenvironment in GBM [6]. Previous work also highlights the promoting role of microRNAs (miRNAs) delivered by GBM-EVs in M2 macrophage polarization [7]. However, the miRNAs derived from GBM-EVs are not fully understood, and it is necessary to further explore the detailed function of GBM-EVs carrying miRNAs in M2 macrophage polarization.

miRNAs can be EVs’ components playing a signaling role in the progression of cancers [8]. The potential role of miR-27a-3p has been documented in the glioma development [9]. Furthermore, hsa-miR-27a-3p was enriched in circulating EVs [10]. However, few studies analyzed the functional roles of hsa-miR-27a-3p derived from GBM-EVs on GBM and the relevant tumor microenvironment. Additionally, enhancer of *zeste homologue 1* (*EZH1*) is strikingly downregulated in GBM [11]. What’s more, *EZH1* is able to suppress the polarization of M2 macrophage [12]. We attempted to elaborate the relationship between hsa-miR-27a-3p carried in GBM-EVs and the underlying regulatory mechanism to provide better understanding of GBM and gain functional insights into the GBM-EVs mediated miRNAs.

## Results

### GBM-EVs induce M2 macrophage polarization under hypoxia condition

**Fig. 1:**
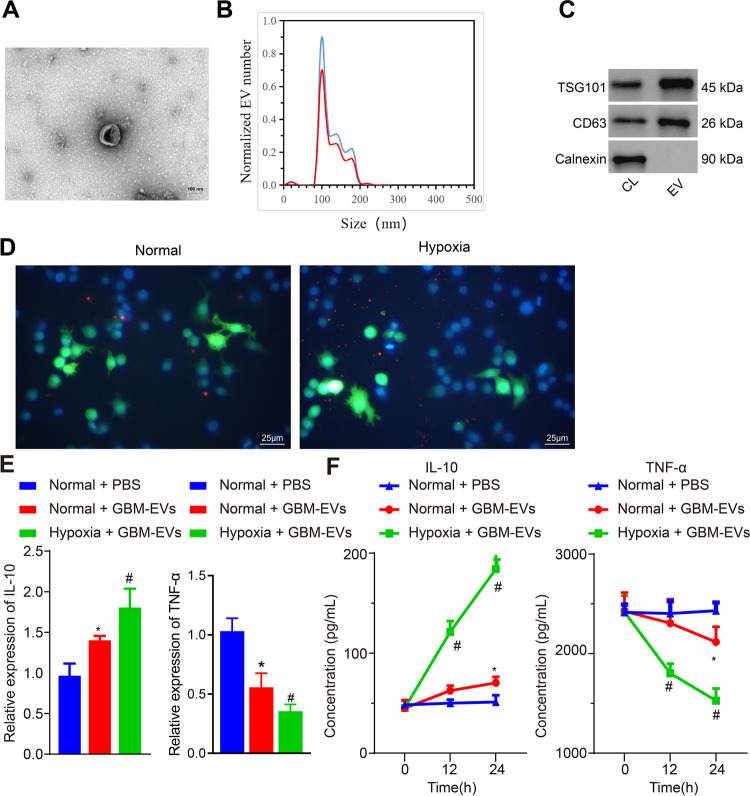
GBM-EVs promote M2 polarization. **A** TEM results of GBM-EVs (scale bar = 100 nm). **B** NTA results of the size distribution of EVs. **C** Expression of marker proteins TSG101, CD63 and Calnexin in 5 μg GBM-EVs and 20 μg cell lysates determined using Western blot analysis. **D** Immunofluorescence images of EV uptake by macrophages under normoxia/hypoxia (double label; red represents EVs; green indicates macrophage) (scale bar = 25 μm). **E** RT-qPCR results of *IL-10* and *TNF-α* mRNA expression in macrophages. **F** ELISA results of the secretion levels of IL-10 and TNF-α in the supernatant of macrophages. *p < 0.05 compared with the normal cells treated with PBS. # *p* < 0.05 compared with normal cells treated with GBM-EVs. The experiment was repeated 3 times independently.

To explore whether GBM-EVs promote M2 macrophage polarization under hypoxia condition, GBM-EVs were extracted from the supernatant of cultured human GBM cell line U87MG, and further identified by transmission electron microscope (TEM), nano-particle tracking analysis (NTA) and Western blot analysis. The results showed that GBM-EVs were typical round particles expressing CD63 and TSG101 proteins instead of calnexin proteins, with an average diameter of 103 ± 6.1 nm (Fig. 1A–C).

Meanwhile, human monocyte cell line THP-1 was treated with phorbol-myristate-acetate (PMA) to induce the differentiation into macrophages. Following that, macrophages were stained with carboxyfluorescein succinimidyl ester (CFSE) while GBM-EVs were stained with deep red staining solution. Subsequently, the stained GBM-EVs were co-cultured with CFSE-stained macrophages under normoxia or hypoxia conditions, respectively. After 24-h co-culture, it was found through the fluorescence microscope that macrophages internalized GBM-EVs, and more GBM-EVs were internalized by macrophages under hypoxia condition (Fig. 1D).

As measured by reverse transcription quantitative polymerase chain reaction (RT-qPCR) and Enzyme-linked immunosorbent assay (ELISA), GBM-EVs under hypoxic significantly increased the expression (Normal + GBM-EVs *vs*. Normal + PBS, 1.4-fold, *p* = 0.04, *n* = 3; Hypoxia + GBM-EVs *vs*. Normal + GBM-EVs, 1.8-fold, *p* < 0.01, *n* = 3) and secretion (Normal + GBM-EVs *vs*. Normal + PBS, 1.3-fold, *p* = 0.02, *n* = 3; Hypoxia + GBM-EVs *vs*. Normal + GBM-EVs, 3.6-fold, *p* < 0.01, *n* = 3) of interlukin-10 (IL-10) in macrophages, but prominently reduced the expression (Normal + GBM-EVs *vs*. Normal + PBS, 0.5-fold, *p* = 0.02, *n* = 3; Hypoxia + GBM-EVs *vs*. Normal + GBM-EVs, 0.3-fold, *p* < 0.01, *n* = 3) and secretion (Normal + GBM-EVs *vs*. Normal + PBS, 0.8-fold, *p* = 0.04, *n* = 3; Hypoxia + GBM-EVs *vs*. Normal + GBM-EVs, 0.6-fold, *p* < 0.01, *n* = 3) of tumor necrosis factor-α (TNF-α) (Figs. 1E, F), confirming that GBM-EVs induced M2 macrophage polarization under hypoxic condition than normoxic condition.

### hsa-miR-27a-3p is transferred from GBM cells to macrophages via EVs

**Fig. 2:**
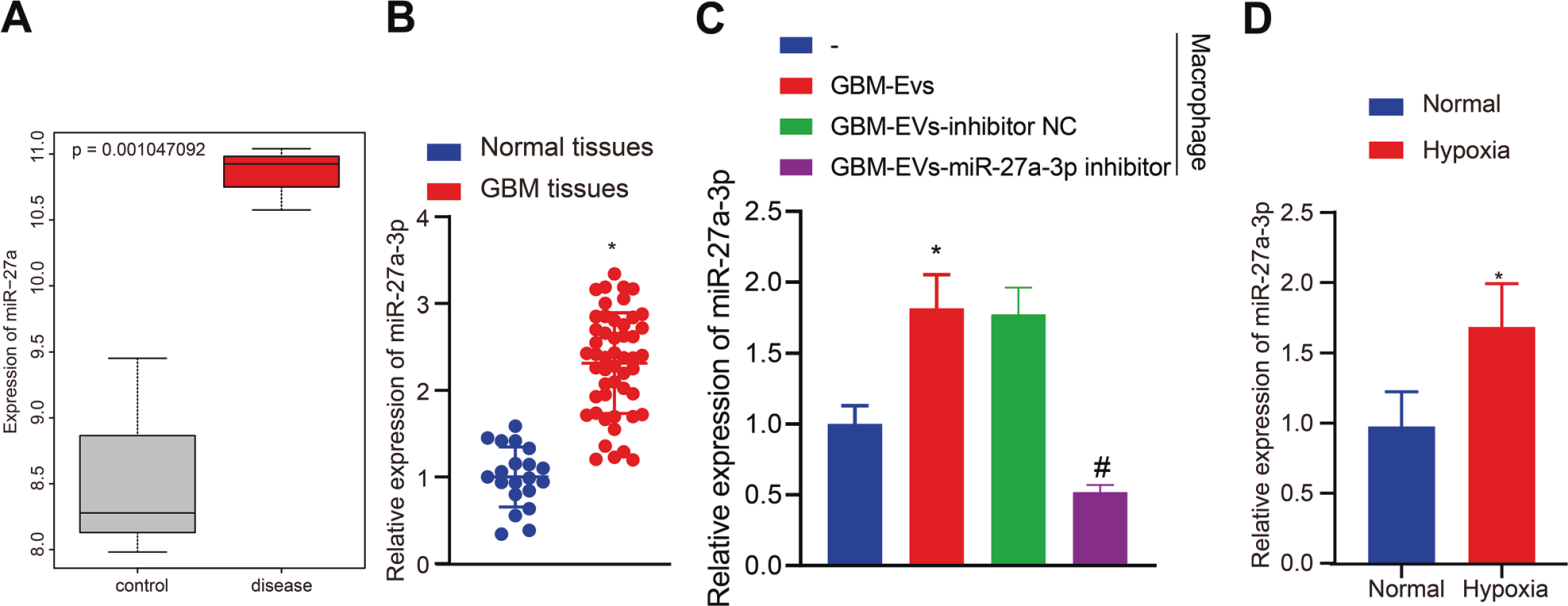
hsa-miR-27a-3p is transferred into macrophages from GBM cells via EVs. **A** Differential expression of hsa-miR-27a-3p in microarray GSE65626. The X-axis represents sample type, and the Y-axis represents expression value. Gray box indicates normal samples (*n* = 3), while red box indicates tumor samples (*n* = 3). **B** RT-qPCR results of hsa-miR-27a-3p expression in clinical samples of patients with GBM (*n* = 50) and non-GBM patients (*n* = 20). **C** RT-qPCR results of hsa-miR-27a-3p expression in macrophages exposed to GBM-EVs relative to normal macrophages. **D** RT-qPCR results of hsa-miR-27a-3p expression in macrophages cultured under normoxia/hypoxia conditions following co-culture with GBM-EVs. *p < 0.05 compared with normal tissues, normal cells or macrophages treated with mimic-NC. #*p* < 0.05 compared with macrophages treated with GBM-EVs-inhibitor NC. The experiment was repeated 3 times independently.

Furthermore, elevated hsa-miR-27a-3p expression was found in GBM-related microarray GSE65626 (*p* < 0.01, Fig. 2A), which was also confirmed by RT-qPCR determination in clinical specimens (2.3-fold, *p* < 0.01, Fig. 2B). Additionally, elevated hsa-miR-27a-3p expression was observed in macrophages exposed to GBM-EVs relative to normal macrophages (Macrophage with GBM-EVs *vs*. Macrophage, 1.6-fold, *p* < 0.01, *n* = 3) while diminished hsa-miR-27a-3p expression was detected in macrophages exposed to GBM-EVs treated with hsa-miR-27a-3p inhibitor (Macrophage with GBM-EVs-hsa-miR-27a-3p inhibitor *vs*. Macrophage with GBM-EVs-inhibitor NC, 0.3-fold, *p* < 0.01, *n* = 3) (Fig. 2C). We further found from RT-qPCR that compared with macrophages under normoxia, hsa-miR-27a-3p showed higher expression in macrophages under hypoxia (Hypoxia *vs*. Normal, 1.7-fold, *p* = 0.04, *n* = 3, Fig. 2D).

Together with the results shown in Fig. 1D, we concluded that hsa-miR-27a-3p was delivered to macrophages by GBM-EVs.

### hsa-miR-27a-3p induces M2 macrophage polarization to promote GBM cell proliferation and motility

Subsequently, miR-27a-3p mimic/miR-27a-3p inhibitor was delivered into macrophages induced by PMA from THP1 cells to demonstrate the action of hsa-miR-27a-3p in macrophage polarization, followed by culturing under normoxia/hypoxia. RT-qPCR determination showed increased IL-10 but decreased TNF-α after treatment of hsa-miR-27a-3p mimic under hypoxia/normoxia, while hsa-miR-27a-3p inhibitor induced the opposite effect (*p* < 0.05, *n* = 3, Fig. S1A–1C). In addition, determination of expression of macrophage polarization marker inducible NOS (iNOS) and arginase-1 (Arg-1) revealed that iNOS was downregulated and Arg-1 was upregulated in macrophages transfected with hsa-miR-27a-3p mimic under hypoxia/normoxia, while the opposite effects were observed in macrophages transfected with hsa-miR-27a-3p inhibitor (*p* < 0.05, *n* = 3, Fig. S1D, 1E). The findings concluded that hsa-miR-27a-3p induced M2 macrophage polarization under hypoxia/normoxia.

Next, the proliferation ability of GBM cells co-cultured with macrophages was strengthened after hsa-miR-27a-3p mimic treatment (1.4-fold, *p* < 0.01, *n* = 3), but weakened in GBM cells co-cultured with hsa-miR-27a-3p inhibitor-treated macrophages (1.7-fold, *p* < 0.01, *n* = 3) (Fig. S1F). Further CFSE staining assay revealed increased proliferating cell ratio of GBM cells co-cultured with hsa-miR-27a-3p mimic-treated macrophages (1.7-fold, *p* < 0.01, *n* = 3), while proliferating cell ratio of GBM cells was found to be diminished after co-culture with hsa-miR-27a-3p inhibitor-treated macrophages (5.8-fold, *p* < 0.01, *n* = 3) (Fig. S2A).

In addition, the results of Transwell assay showed higher migration and invasion of GBM cells co-cultured with hsa-miR-27a-3p mimic-treated macrophages, while cell migration and invasion was curtailed in GBM cells co-cultured with hsa-miR-27a-3p inhibitor-treated macrophages (*p* < 0.01, *n* = 3) (Fig. S1G).

Thus, hsa-miR-27a-3p promoted the polarization of M2 macrophages, thereby facilitating the GBM cell proliferation and motility.

### *EZH1* is a target gene of hsa-miR-27a-3p

**Fig. 3:**
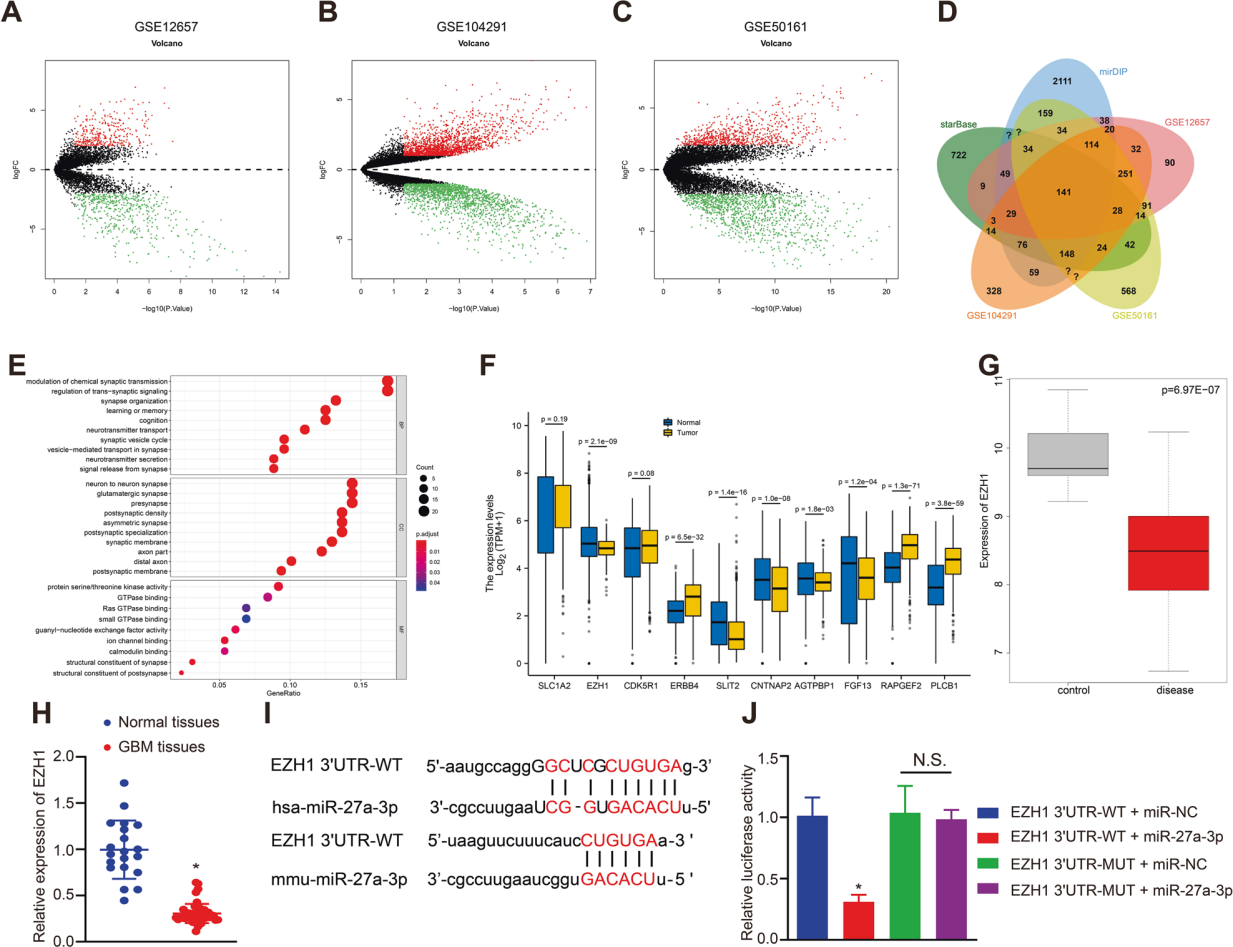
hsa-miR-27a-3p targets EZH1. **A** Volcano plots displaying the differentially expressed genes retrieved from microarray dataset GSE12657. **B** Volcano plots displaying the differentially expressed genes retrieved from microarray dataset GSE104291. **C** Volcano plots displaying the differentially expressed genes retrieved from microarray dataset GSE50161. The X-axis represents -log10 (*p* value), the Y-axis represents logFoldChange, red dots represent upregulated genes, and green dots indicate downregulated genes. **D** Venn diagram displaying the intersection of target genes of hsa-miR-27a-3p predicted by bioinformatics analysis and differentially expressed genes retrieved from microarray datasets GSE12657, GSE104291 and GSE50161. **E** GO functional enrichment analysis on the intersected genes. X-axis represents gene ratio. Y-axis represents GO entries. The right histogram is color gradation. **F** Expression level of candidate genes in TCGA_LGG and GTEx. **G** The expression of EZH1 in microarray GSE50161. **H** The expression of EZH1 in clinical samples of patients with GBM (*n* = 50) and non-GBM patients (*n* = 20), *p < 0.05 compared with normal tissues. **I** The binding sites between hsa-miR-27a-3p and EZH1 3’UTR in human and mice predicted by starBase databse. **J** The targeting relationship between miR-27a and EZH1 verified by dual luciferase reporter gene assay, *p < 0.05 compared with EZH1 3’UTR-WT + mimic-NC group. The experiment was repeated 3 times independently.

We further predicted the downstream target genes of hsa-miR-27a-3p through starBase and mirDIP databases, revealing 4365 and 6224 genes respectively. Meanwhile, differential analysis was performed on the microarrays GSE12657, GSE104291 and GSE50161 using “limma” package of R language (|logFoldChange| > 1, *p* < 0.05), revealing 864, 3628 and 1797 differentially expressed genes respectively (Fig. 3A–C). After intersection, 141 candidate genes were obtained (Fig. 3D, Table S1) followed by heatmap analysis of their expression in three microarrays (Fig. S3). Subsequently, Gene Ontology (GO) functional analysis of intersected genes revealed that those genes were mainly enriched in the pathways of “modulation of chemical synaptic transmission”, “neuron to neuron synapase” and “protein serine/threonine kinase activity” (Fig. 3E, Table S2). Among the GO pathway enrichment results, pathways related to brain development commanded our attention, including telencephalon development, forebrain development and pallium development. Genes involved in the pathways consisted of *SLC1A2, EZH1, CDK5R1, ERBB4, PLCB1, SLIT2, CNTNAP2, AGTPBP1, FGF13*, and *RAPGEF2*. The candidate genes were checked for differential expression in TCGA_LGG and GTEx (Fig. 3F), results of which showed that *EZH1*, *SLIT2*, *CNTNAP2, AGTPBP1* and *FGF13* were significantly downregulated.

*EZH1* expression was then analyzed in the three GBM-related microarray dataset GSE50161, showing that *EZH1* expression was reduced in GBM (Fig. 3G). RT-qPCR data was confirmatory, showing a decrease in *EZH1* expression in patients with GBM (3.3-fold, *p* < 0.01) (Fig. 3H).

According to the prediction of starBase, there existed binding sites between hsa-miR-27a-3p/mmu-miR-27a-3p and *EZH1* (Fig. 3I), which was verified using luciferase assay that the luciferase intensity of the cells co-transfected with hsa-miR-27a-3p mimic and EZH1 3’untranslated region (3’UTR)-wild type (WT) decreased significantly (*p* < 0.01, *n* = 3) yet that of the cells co-transfected with hsa-miR-27a-3p mimic and EZH1 3’UTR-mutant type (MUT) did not differ significantly (*p* > 0.05, *n* = 3) (Fig. 3J).

Taken together, these findings indicated that *EZH1* was poorly expressed in GBM and hsa-miR-27a-3p targeted *EZH1*.

### GBM-EV-hsa-miR-27a-3p downregulates EZH1 to promote M2 macrophage polarization in contribution to GBM cell proliferation and motility

To further validate the regulation of EZH1 by GBM-EV-hsa-miR-27a-3p at the cellular level, U87MG cells were transfected with hsa-miR-27a-3p mimic, followed by the extraction of EVs. Then, EVs were co-cultured with macrophages overexpressing EZH1 under hypoxia. We found an enhancement in hsa-miR-27a-3p and *IL-10* expression but a reduction in *EZH1* and *TNF-α* expression in the hsa-miR-27a-3p mimic-treated macrophages. However, the expression of hsa-miR-27a-3p did not significantly change in the macrophages co-transfected with hsa-miR-27a-3p mimic and *EZH1* overexpression vector (oe-EZH1) and macrophages co-transfected with hsa-miR-27a-3p and oe-NC, while among hsa-miR-27a-3p mimic- and oe-EZH1-treated cells, the expression of *EZH1* and *TNF-α* increased and *IL-10* expression decreased (*p* < 0.05, *n* = 3) (Fig. S4A–C). In addition, iNOS expression was decreased (2.3-fold, *p* < 0.01, *n* = 3) and Arg-1 expression was increased (1.9-fold, *p* < 0.01, *n* = 3) after the treatment of hsa-miR-27a-3p mimic, while the overexpression of EZH1 reversed the effects of hsa-miR-27a-3p mimic (Fig. S4D). The findings suggested that GBM-EV-hsa-miR-27a-3p induced M2 macrophage polarization by downregulating *EZH1*.

Further CFSE staining assay showed that proliferating cell ratio was increased in presence of hsa-miR-27a-3p mimic (1.7-fold, *p* < 0.01, *n* = 3) yet decreased by additional delivery of oe-EZH1 (1.6-fold, *p* = 0.02, *n* = 3) (Fig. S2B). Then, the proliferative, migrative and invasive capabilities of GBM cells were increased after hsa-miR-27a-3p mimic, but were distinctly suppressed after the addition of oe-EZH1, indicating that the overexpressed EZH1 offset the effects of overexpressed hsa-miR-27a-3p on the proliferation, migration and invasion of GBM cells (*p* < 0.05, *n* = 3) (Figs. S4E, F).

The aforementioned results demonstrated that EVs-hsa-miR-27a-3p induced M2 macrophage polarization by targeting *EZH1* in contribution to GBM cell proliferation, migration and invasion.

### EZH1 inhibits M2 macrophage polarization by suppressing KDM3A expression

**Fig. 4:**
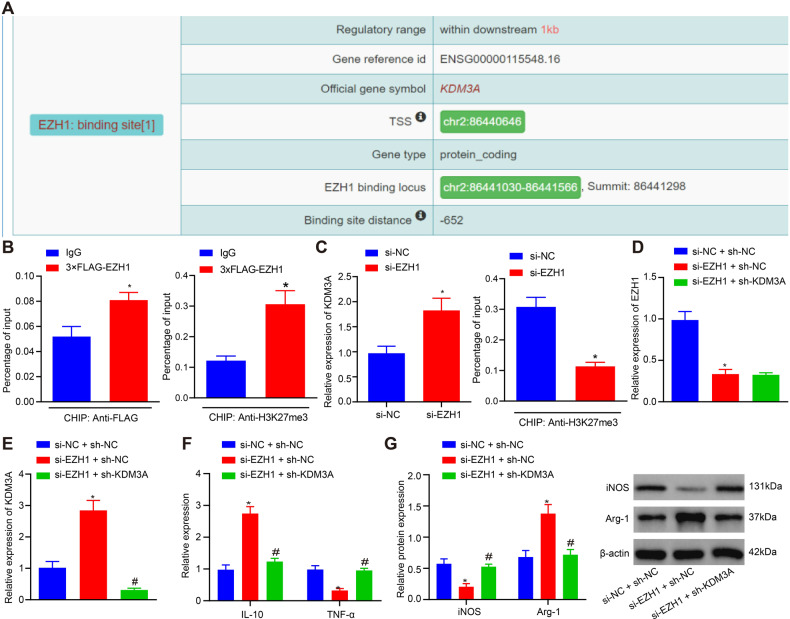
*EZH1* suppresses M2 polarization by downregulating *KDM3A*. **A** ChIP-Seq data analysis results of the binding sites. **B** Enrichment of *EZH1* and H3K27me3 on the *KDM3A* promoter in macrophages detected by ChIP analysis. **C** *KDM3A* mRNA expression in macrophages determined by RT-qPCR and H3K27me3 enrichment on the KDM3A promoter in macrophages detected by ChIP analysis. **D** mRNA expression of *EZH1* in macrophages cultured under hypoxic conditions determined by RT-qPCR. **E** mRNA expression of *KDM3A* in macrophages cultured under hypoxic conditions determined by RT-qPCR. **F** mRNA expression of *IL-10* and *TNF-α* in macrophages cultured under hypoxic conditions determined by RT-qPCR. **G** The expression of iNOS and Arg-1 detected by Western blot analysis. *p < 0.05 compared with IgG, si-NC or si-NC + sh-NC. # *p* < 0.05 com*p*ared with si-EZH1 + sh-NC. The experiment was repeated 3 times independently.

As ChIP-Seq data analysis results unraveled, *EZH1* was found to bind to the promoter of KDM3A (Fig. 4A), suggesting that *EZH1* inhibited *KDM3A* at a transcriptional level. Next, we performed chromatin immunoprecipitation (ChIP) analysis to detect whether *EZH1* mediated expression of *KDM3A* through regulation on H3K27me3 enrichment on the promoter. It was found that overexpressed *EZH1* significantly enhanced H3K27me3 enrichment on the promoter region of *KDM3A* (1.5-fold, *p* < 0.01, *n* = 3) (Fig. 4B). Moreover, short interfering RNA (siRNA) against *EZH1* (si-EZH1) significantly diminished H3K27me3 enrichment on the promoter region of *KDM3A* gene (0.4-fold, *p* < 0.01, *n* = 3) (Fig. 4C).

To further explore the regulatory relationship between *EZH1* and *KDM3A*, *EZH1* or/and *KDM3A* was knocked down in macrophages. Results of RT-qPCR displayed that silencing EZH1 alone upregulated *KDM3A* and *IL-10* but decreased *TNF-α* expression to induce M2 macrophage polarization. While knockdown of *KDM3A* reversed the effects of silenced *EZH1* on M2 macrophage polarization when co-transfected with si-EZH1 (*p* < 0.01, *n* = 3) (Fig. 4D–F). Additionally, the results of Western blot analysis exhibited that silencing *EZH1* downregulated the expression of iNOS (0.4-fold, *p* < 0.01, *n* = 3) and upregulated the expression of Arg-1 (2.0-fold, *p* < 0.01, *n* = 3), while *KDM3A* knockdown reversed the effects of silenced *EZH1* on M2 polarization (Fig. 4G).

These results suggested that *EZH1* inhibited M2 macrophage polarization by inhibiting *KDM3A* expression through H3K27me3 enrichment.

### *KDM3A* facilitates macrophage polarization by upregulating CTGF

**Fig. 5:**
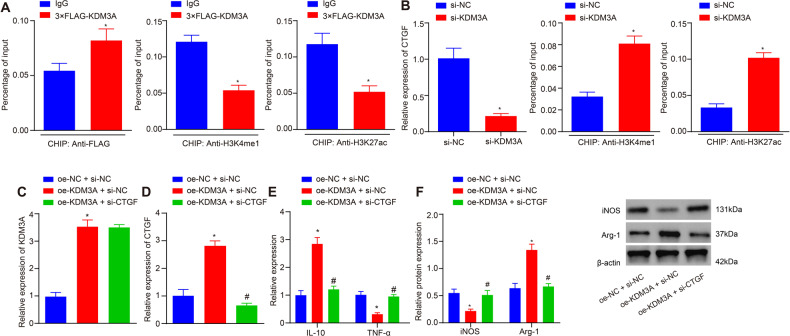
*KDM3A* upregulates *CTGF* expression to boost M2 polarization. **A** ChIP analysis results on enrichment of H3K4me1 and H3K27ac in the CTGF enhancer region of macrophages after addition of KDM3A. **B** ChIP analysis results on enrichment of H3K4me1 and H3K27ac in the CTGF enhancer region of macrophages after knockdown of KDM3A. **C** mRNA expression of *KDM3A* in macrophages cultured under hypoxic conditions determined by RT-qPCR. **D** mRNA expression of *CTGF* in macrophages cultured under hypoxic conditions determined by RT-qPCR. **E** mRNA expression of *IL-10* and *TNF-α* in macrophages cultured under hypoxic conditions determined by RT-qPCR. **F** Western blot analysis results of the protein expression of iNOS and Arg-1. *p < 0.05 compared with IgG, si-NC or oe-NC + si-NC. #*p* < 0.05 com*p*ared with oe-KDM3A + si-NC. The experiment was repeated 3 times independently.

ChIP analysis was then performed using antibodies to CTGF, H3K27ac and H3K4me1 after treatment of macrophages with si-KDM3A. It was found that KDM3A protein was highly enriched in the enhancer region of CTGF gene (1.5-fold, *p* = 0.02, *n* = 3) and inhibited the enrichment of H3K4me1 (0.4-fold, *p* < 0.05, *n* = 3) and H3K27ac (0.4-fold, *p* < 0.05, *n* = 3) in the enhancer region (Fig. 5A, B). Also, knockdown of *KDM3A* reduced *CTGF* expression level (0.2-fold, *p* < 0.05, *n* = 3) (Fig. 5B).

To further explore the regulatory relationship between *KDM3A* and *CTGF*, *KDM3A* was overexpressed or/and *CTGF* was knocked down in macrophages. Results of RT-qPCR exhibited that overexpression of *KDM3A* upregulated *CTGF* (2.7-fold, *p* < 0.05, *n* = 3) and *IL-10* (2.8-fold, *p* < 0.05, *n* = 3) expression but decreased *TNF-α* expression (0.3-fold, *p* < 0.05, *n* = 3). However, after overexpressing *KDM3A* or knocking *CTGF* down in macrophages, it was found that depleted *CTGF* inhibited M2 macrophage polarization which was induced by overexpressed *KDM3A* (Fig. 5C–E). Moreover, overexpression of *KDM3A* downregulated iNOS protein expression (0.4-fold, *p* < 0.05, *n* = 3) and upregulated Arg-1 protein expression (2.1-fold, *p* < 0.05, *n* = 3), while knockdown of *CTGF* reversed the M2 macrophage polarization induced by overexpression of *KDM3A* (Fig. 5F).

These results suggested that *KDM3A* promoted M2 macrophage polarization by binding to *CTGF* enhancer regions and promoting *CTGF* gene expression through inhibition on enrichment of H3K4me1 and H3K27ac.

### GBM-EVs-released hsa-miR-27a-3p induces M2 macrophage polarization to promote GBM cell proliferation, migration and invasion by *EZH1/KDM3A/CTGF* in vitro

**Fig. 6:**
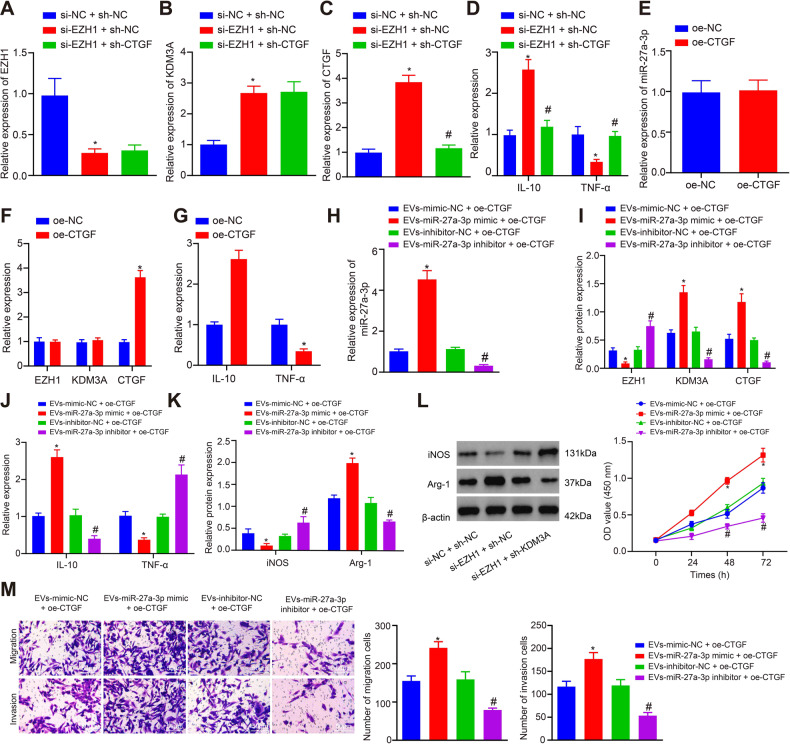
GBM-EV-derived hsa-miR-27a-3p targets *EZH1* to mediate M2 polarization through KDM3A/CTGF in vitro. **A** mRNA expression of *EZH1* in macrophages after *EZH1* and *CTGF* knockdown determined using RT-qPCR. **B** mRNA expression of *KDM3A* in macrophages after *EZH1* and *CTGF* knockdown determined using RT-qPCR. **C** mRNA expression of *CTGF* in macrophages after *EZH1* and *CTGF* knockdown determined using RT-qPCR. **D** mRNA expression of *IL-10* and *TNF-α* in macrophages after *EZH1* and *CTGF* knockdown determined using RT-qPCR. **E** mRNA expression of *EZH1* in macrophages overexpressing *CTGF* evaluated using RT-qPCR. **F** mRNA expression of *KDM3A* in macrophages overexpressing *CTGF* evaluated using RT-qPCR. **G** mRNA expression of *CTGF* in macrophages overexpressing *CTGF* evaluated using RT-qPCR. **H** Expression of hsa-miR-27a-3p after macrophages were transfected with hsa-miR-27a-3p mimic/inhibitor determined using RT-qPCR. **I** Protein expression of EZH1, KDM3A and CTGF in macrophages measured using Western blot analysis. **J** mRNA expression of *IL-10* and *TNF-α* in macrophages detected using RT-qPCR. **K** Protein expression of iNOS and Arg-1 in macrophages determined using Western blot analysis. **L** CCK-8 assay results of proliferation ability of GBM cells. **M** Transwell assay results of the migration and invasion ability of GBM cells (scale bar = 50 μm). *p < 0.05 compared with si-NC + sh-NC, oe-NC or oe-CTGF + EVs-mimic-NC. #*p* < 0.05 com*p*ared with si-EZH1 + sh-NC or oe-CTGF + EVs-inhibitor-NC. The experiment was repeated 3 times independently.

To determine whether *EZH1* can regulate *CTGF* expression through *KDM3A*, *EZH1* or *CTGF* was knocked down in macrophages, followed by the determination of *EZH1*, *KDM3A* and *CTGF* expression. We found decreased *EZH1* and *TNF-α* expression but upregulated *KDM3A*, *CTGF* and *IL-10* after *EZH1* was silenced. However, the expression of *EZH1* and *KDM3A* did not change significantly in the si-EZH1 + sh-CTGF group compared with the si-EZH1 + sh-NC group, while the expression of CTGF (0.3-fold, *p* < 0.05, *n* = 3) and IL-10 (0.5-fold, *p* < 0.05, *n* = 3) decreased after *EZH1* and *CTGF* were both silenced, along with increased *TNF-α* expression (2.9-fold, *p* < 0.05, *n* = 3), indicating that knockdown of *CTGF* reversed the M2 macrophage polarization induced by overexpressed *KDM3A* and silenced *EZH1* (Fig. 6A–D).

In order to further determine that GBM-EVs-derived hsa-miR-27a-3p functioned as an upstream regulator of *EZH1/KDM3A/CTGF* in M2 macrophage polarization, *CTGF* was overexpressed in macrophages, followed by the determination of hsa-miR-27a-3p/*EZH1/KDM3A/CTGF* expression in each group. We observed that oe-CTGF exerted no effect on the expression of hsa-miR-27a-3p, *EZH1*, and *KDM3A*. However, *CTGF* and *IL-10* expression was increased and *TNF-α* expression was decreased after *CTGF* was overexpressed (Fig. 6E–G). EVs were extracted from hsa-miR-27a-3p mimic/inhibitor treated GBM cells, which were co-cultured with oe-CTGF-treated macrophages. The results showed that in EVs extracted from hsa-miR-27a-3p mimic-treated GBM cells, upregulated *CTGF* prominently stimulated M2 macrophage polarization, in which the expression of hsa-miR-27a-3p (4.5-fold, *p* < 0.05, *n* = 3), *KDM3A* (2.2-fold, *p* < 0.05, *n* = 3), *IL-10* (2.6-fold, *p* < 0.05, *n* = 3) and *CTGF* (2.2-fold, *p* < 0.05, *n* = 3) increased, but *EZH1* (0.3-fold, *p* < 0.05, *n* = 3) and *TNF-α* (0.4-fold, *p* < 0.05, *n* = 3) expression reduced. In contrast, in the EVs extracted from hsa-miR-27a-3p inhibitor-treated cells, upregulated *CTGF* by oe-CTGF dampened M2 macrophage polarization accompanied with downregulated hsa-miR-27a-3p (0.3-fold, *p* < 0.05, *n* = 3), *KDM3A* (0.2-fold, *p* < 0.05, *n* = 3)*, CTGF* (0.2-fold, *p* < 0.05, *n* = 3) and *IL-10* (0.4-fold, *p* < 0.05, *n* = 3) expression but upregulated *EZH1* (2.3-fold, *p* < 0.05, *n* = 3) and *TNF-α* expression (2.2-fold, *p* < 0.05, *n* = 3) (Fig. 6H–J). Furthermore, expression of iNOS decreased (0.4-fold, *p* < 0.05, *n* = 3) and Arg-1 increased (2.2-fold, *p* < 0.05, *n* = 3) in the GBM-EVs containing hsa-miR-27a-3p mimic and macrophages overexpressing *CTGF*. However, the results were reversed in the GBM-EVs containing hsa-miR-27a-3p inhibitor and macrophages overexpressing *CTGF* (iNOS: 2.2-fold, *p* < 0.05, *n* = 3; Arg-1: 0.4-fold, *p* < 0.05, *n* = 3) (Fig. 6K).

The role of conditioned macrophages in the GBM cell biological processes was further explored in vitro. We observed that in GBM-EVs containing hsa-miR-27a-3p mimic and macrophages overexpressing *CTGF*, strengthened proliferation (1.5-fold, *p* < 0.05, *n* = 3), invasion (1.5-fold, *p* < 0.05, *n* = 3) and migration (1.6-fold, *p* < 0.05, *n* = 3) abilities was observed, which was abolished in GBM-EVs containing hsa-miR-27a-3p inhibitor and macrophages overexpressing *CTGF* (proliferation: 2.0-fold, *p* < 0.05, *n* = 3; migration: 2.0-fold, *p* < 0.05, *n* = 3; invasion: 2.2-fold, *p* < 0.05, *n* = 3) (Fig. 6L, M).

Thus, GBM-EVs carrying hsa-miR-27a-3p boosted M2 macrophage polarization and ultimately facilitated the proliferative, migrative and invasive capabilities of GBM cells via the *EZH1/KDM3A/CTGF* axis.

### GBM-EV-derived hsa-miR-27a-3p induces M2 macrophage polarization to aggravate GBM progression in vivo

**Fig. 7:**
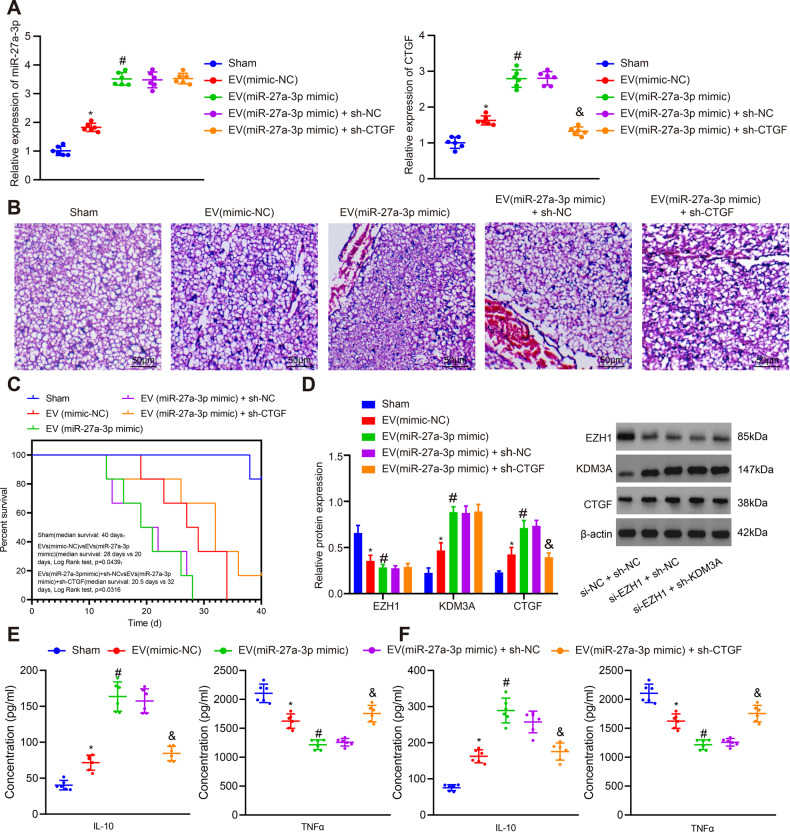
GBM-EV-derived hsa-miR-27a-3p aggravates GBM in vivo. Nude mice were injected with EVs from GBM cells overexpressing hsa-miR-27a-3p or/and macrophages transfected with lentivirus-mediated *CTGF*. **A** hsa-miR-27a-3p expression and *CTGF* mRNA expression in each group of mice (*n* = 6) determined using RT-qPCR. **B** H&E staining results of GBM cell invasion to the mouse brain tissues (*n* = 6) (scale bar = 50 μm). **C** Statistical data of survival time of mice in each group (*n* = 6). **D** Western blot analysis results of the protein expression of EZH1, KDM3A and CTGF in mouse brain tissues of each group (*n* = 6). **E** ELISA results of secretion levels of IL-10 and TNF-α in peripheral serum of mice (*n* = 6). **F** ELISA results of content levels of IL-10 and TNF-α in lysate of mouse tumor xenografts (*n* = 6). *p < 0.05 compared with sham-operated mice. #*p* < 0.05 compared with mice harboring EVs containing mimic-NC. & *p* < 0.05 compared with mice harboring EVs containing hsa-miR-27a-3p mimic + sh-NC.

To validate the above-mentioned results in vivo, nude mice were injected with EVs from GBM cells overexpressing hsa-miR-27a-3p or/and macrophages transfected with lentivirus-mediated *CTGF* to develop xenograft tumors. The results of RT-qPCR showed higher transfection efficiency of hsa-miR-27a-3p and *CTGF* (*p* < 0.01, *n* = 6) (Fig. 7A).

Hematoxylin-Eosin (H&E) staining exhibited that the boundary of mouse brain tissues was blurred after treatment of GBM-EVs harboring hsa-miR-27a-3p mimic, accompanied with elevated invasive ability of GBM cells. While the invasive ability of tumors to brain tissues was weaker in mice harboring hsa-miR-27a-3p mimic-treated GBM-EVs after *CTGF* expression was silenced (Fig. 7B). In addition, the survival time of mice was shortened after GBM-EVs overexpressing hsa-miR-27a-3p were injected into mice, while the survival time of mice was prolonged after interfering with *CTGF* (Fig. 7C).

Additionally, GBM-EVs carrying hsa-miR-27a-3p mimic led to downregulated EZH1 (0.8-fold, *p* = 0.03, *n* = 6) and upregulated KDM3A (1.9-fold, *p* < 0.01, *n* = 6) and CTGF (1.7-fold, *p* < 0.01, *n* = 6) in mouse brain tissues. However, after silencing of CTGF, the expression of EZH1 and KDM3A did not change significantly, while the expression of CTGF was downregulated (0.5-fold, *p* < 0.01, *n* = 6) (Fig. 7D). Moreover, GBM-EVs carrying overexpressed hsa-miR-27a-3p resulted in the increase of IL-10 (2.3-fold, *p* < 0.01, *n* = 6) and the decrease of TNF-α (0.5-fold, *p* < 0.01, *n* = 6) in peripheral serum of mice, indicating that M2 polarization was induced in mice. However, the decrease of IL-10 (0.7-fold, *p* < 0.01, *n* = 6) and the increase of TNF-α (1.4-fold, *p* < 0.01, *n* = 6) were observed after silencing of CTGF, suggesting that loss of CTGF prevented M2 macrophages polarization to some degree (Fig. 7E). In addition, ELISA in tumor xenograft revealed upregulation of IL-10 (1.8-fold, *p* < 0.01, *n* = 6) and downregulation of TNF-α (0.7-fold, *p* < 0.01, *n* = 6) in presence of hsa-miR-27a-3p mimic while additional treatment of short hairpin RNA (shRNA) against CTGF (sh-CTGF) led to a reduction in IL-10 (0.7-fold, *p* < 0.01, *n* = 6) and an increase in (1.6-fold, *p* < 0.01, *n* = 6) (*p* < 0.01, *n* = 6) (Fig. 7F).

These results suggested that GBM-EVs-derived hsa-miR-27a-3p promoted the development of GBM in vivo by induces M2 macrophage polarization.

## Discussion

EVs activating macrophages is now considered as key players in cancer progression, as macrophages are able to promote the growth of tumors [13]. Moreover, hypoxia condition is likely to stimulate GBM cells to secrete EVs, hence promoting the GBM cell motility [14]. This study investigated the effects of hsa-miR-27a-3p derived from GBM-EVs on M2 macrophage polarization. We demonstrated that GBM-EVs delivered hsa-miR-27a-3p to participate in the polarization of M2 macrophage.

We initially revealed that GBM-EVs delivered hsa-miR-27a-3p, highly expressed in GBM tissues and cells, to polarize M2 macrophage. Upregulation of miR-27a-3p has also been detected in human neoplastic brain tissues in contribution to glioma cell proliferation [15]. Coincidentally, EVs derived from monocytes containing miR-27a also play promoting role in the polarization of M2 macrophages [16]. Moreover, miR-27a that enhances proliferation and migration of GBM cells is abundant in GBM-EVs [17]. These works further support our statement that hsa-miR-27a-3p contained in GBM-EVs polarized M2 macrophage. Furthermore, hsa-miR-27a-3p expression was silenced by using hsa-miR-27a-3p inhibitor. We found that loss of hsa-miR-27a-3p upregulated the protein level of iNOS but downregulated Arg-1. It is reported that iNOS is one of the phenotypes of M1 macrophage while Arg-1 is a marker for M2 macrophage [18]. Likewise, miR-27a-3p in EVs from mesenchymal stem cells has been demonstrated as a key regulator of M2 macrophage polarization to alleviate acute lung injury [19]. Therefore, downregulated hsa-miR-27a-3p curtailed the M2 macrophage polarization.

We next found that hsa-miR-27a-3p suppressed *EZH1* expression, the target of hsa-miR-27a-3p. *EZH1* is reported to participate in the macrophage phenotype shifting and downregulation of *EZH1* is able to promote M2 macrophage [12]. To our best knowledge, the relationship between hsa-miR-27a-3p and *EZH1* is barely explored in previous literature. Furthermore, we found that *EZH1* participated in the regulation of M2 polarization in association with *KDM3A*. Prior work also demonstrates that EZH1, as a H3K27me3 methylase, binds to the downstream gene promoter and promotes H3K27me3 to suppress gene expression [20], which is in line with our study proposing that *EZH1* inhibited the expression of *KDM3A*. As prior work verified, M2 macrophage can be induced by *KDM3A* [21]. Additionally, it is addressed that *KDM3A*, a demethylase of histone H3K9me1/2, can promote the expression of *CTGF* by facilitating H3K27ac on the enhancers of *CTGF* [22]. While *CTGF* promotes drug-resistance in GBM cells and facilitates the progression of GBM [23]. Based on what has been discussed above, it was indicated that hsa-miR-27a-3p secreted from GBM-EVs downregulated *EZH1* expression, elevated expression of *KDM3A* and further upregulated CTGF to polarize M2 macrophage.

In vitro analysis further unraveled that proliferative, migrative and invasive capabilities of GBM cells were expedited by overexpressed hsa-miR-27a-3p and the underlying mechanism. Promoted M2 macrophage is associated with the enhanced proliferation and migration abilities of GBM cells [24, 25]. Largely in agreement with our finding, miR-1246 encapsulated in EVs derived from glioma cells under hypoxic condition has been deciphered to trigger M2 macrophage polarization [7]. To confirm our results in vitro, we also employed murine GBM cell line GL261 for generating xenograft mouse models to study the effects of hsa-miR-27a-3p/*EZH1/KDM3A/CTGF* on development of GBM in vivo. We concluded that overexpressed hsa-miR-27a-3p induced upregulated IL-10 and downregulated TNF-α in peripheral serum of mice. Promoted M2 macrophage polarization is related to increased IL-10 and decreased TNF-α [26]. Therefore, the results derived from in vitro analysis were consistent with what we had concluded from in vivo experiment.

**Fig. 8:**
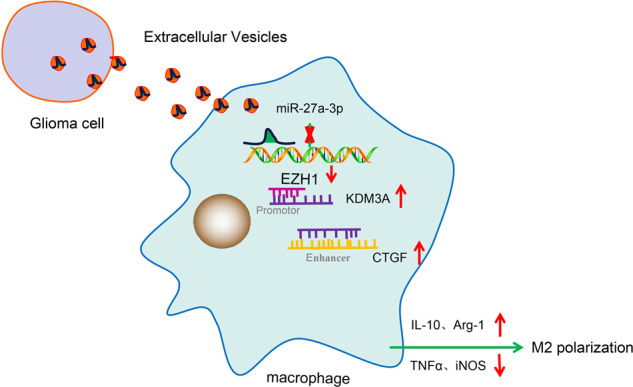
Schematized molecular mechanism underlying hsa-miR-27a-3p contained in GBM-EVs. EV hsa-miR-27a-3p inhibits the mRNA expression of target gene *EZH1* to upregulate *KDM3A*-mediated *CTGF* mRNA expression, which induces M2 macrophage polarization and further facilitates proliferation, migration and invasion of GBM cells.

In summary, our study collaboratively suggests that hsa-miR-27a-3p contained in GBM-EVs inhibits *EZH1* expression to upregulate *KDM3A*-mediated *CTGF* expression, which induces M2 macrophage polarization and further facilitates proliferative, migrative and invasive capabilities of GBM cells (Fig. 8). Our findings show that the GBM-EVs-derived hsa-miR-27a-3p may be a biomarker for diagnosis of GBM. It is likely that downregulated hsa-miR-27a-3p in GBM-EVs served as a tool to combat GBM. Further studies of the molecular mechanisms underlying tumor-associated macrophages *via* the EVs will facilitate better understanding on the effects of GBM-EVs on the progression of GBM.

## Materials and methods

### Ethics statement

The usage of cerebrospinal fluid and tumor tissues was approved by the Institutional review board of the Jilin Medical University. This study was performed according to the *Declaration of Helsinki* and was approved by the ethics committee of Jilin Medical University (2017-018). All patients were informed of the study and tested voluntarily with written informed consents provided. Animal experiments were conducted according to the ethical policies and procedures approved by the ethics committee of the Jilin Medical University (2017-018).

### Bioinformatics analysis

The Gene Expression Omnibus database was used to identify glioma-related miRNA microarray dataset GSE65626 and glioma-related mRNA microarray datasets GSE12657, GSE104291 and GSE50161 through the R package ‘limma’. The differentially expressed genes were screened utilizing |logFoldChange | > 1.0, and adj.*p*.Val <0.05. The downstream target genes of miRNA were predicted through starBase and mirDIP databases. The low grade glioma-related data of TCGA and RNAseq data in the format of transcripts per million reads were obtained by UCSC XENA Toil Recompute Compendium, among which GTEx data was extracted from normal brain tissue data. After log2 processing, genes between normal and tumor tissues were compared and tested by Mann-Whitney U (Wilcoxon rank sum test). *p* < 0.05 was suggestive of significant difference.

### Study subjects

GBM tissue samples were collected from patients with GBM (*n* = 50, age ≤65 years) who underwent surgical treatment in the oncological neurosurgery department of the First Hospital of Jilin University from June 2016 to June 2019. Sample controls were obtained from non-GBM patients (*n* = 20) with encephalopathy.

Patients were enrolled if they were diagnosed as GBM based on clinical routine tests and pathological results without chemoradiotherapy or biological immunotherapy before. Patients were excluded if (1) they had visceral disorders (heart, lung, liver, etc.), autoimmune diseases or other malignancies; (2) they could not communicate because of mental disorders or conscious disturbance; (3) they had undergone chemoradiotherapy before; (4) they had serious complications like intracranial hemorrhage after surgery.

### Cell culture

Human GBM cell line U87MG (CL-0238, Procell Life Science&Technology Co., Ltd.), human monocyte cell line THP-1 (TCHu 57, the Institute of Biochemistry and Cell Biology, Chinese Academy of Sciences) and murine GBM GL261 (BNCC338268, BNCC, Beijing, China) were collected for our study. U87MG cell was cultured in Dulbecco’s modified eagle medium (DMEM; Thermo Fisher Scientific, Waltham, MA) containing 10% fetal bovine serum (FBS). Meanwhile, THP-1 cell was cultured in Roswell Park Memorial Institute-1640 medium (Thermo Fisher Scientific) with 10% FBS, and further incubated with 100 ng/mL PMA (Sigma-Aldrich, St. Louis, MO) for 24 h in vitro to induce the differentiation into macrophages. Additionally, EVs (final concentration: 1 μg/mL) were added to the medium of recipient cells for co-culture. All cell lines were identified by short tandem repeat to be mycoplasma negative before the experiments. miRNA inhibitor/mimic, siRNA against EZH1 and control siRNA were purchased from GenePharma (Shanghai, China).

### Isolation and identification of EVs

U87MG cells were cultured in exosome-depleted DMEM (A2720801, GIBCO BRL, Green Island, NY) containing 10% FBS under normoxia (21% O_2_) or hypoxia (1% O_2_). After 48–72 h of incubation, the culture medium (30 mL) was collected and centrifuged at 300 × *g* for 10 min, 2000 × *g* for 15 min and 12,000 × *g* for 30 min to remove the floating cells and cell debris, followed by filtering through 0.22-μm filter. The supernatant was subjected to centrifugation at 100,000 × *g* for 2 h. After PBS wash, another round of ultracentrifugation was performed at 100,000 × *g* for 2 h. The precipitate was re-suspended in 100 μL PBS and stored at −80 °C for further or immediate use.

Observation of morphological characteristics of EVs was performed under a TEM (JEM-1010; JEOL, Tokyo, Japan). Then, the size was measured using Zetasizer Nano ZS90 instrument (Malvern, UK) with five videos of typically 60 s’ duration taken, and the data was analyzed by Zetasizer software v7.11, Malvern Instruments. The EV surface marker proteins rabbit anti-CD63 (ab134045, 1: 1000, Abcam, Cambridge, UK), rabbit anti-TSG101 (ab125011, 1: 1000, Abcam) and rabbit anti-Calnexin (ab92573, 1: 20000, Abcam) were determined using Western blot analysis.

### EV labeling and macrophage staining

EVs (concentration: 0.1–0.2 μg) were resuspended in 400 μL PBS and stained with CellMask Deep Red (Thermo Fisher Scientific) at excitation/emission wavelengths of 649/666 nm. During the labeling, EVs were incubated with deep red staining solution (1: 1000) for 20 min at 37 °C. The remaining dye liquor was removed by PBS wash (1 to 10,000 v/v ratio). Then, EVs were centrifuged at 100,000 × *g* for 1 h and diluted in PBS, followed by the determination of protein concentration using bicinchoninic acid (BCA) protein detection kit. Cells were stained with CellTrace^TM^ CFSE (Life Technologies, Carlsbad, CA) with a maximum excitation/emission wavelength of 492/517 nm. The immunofluorescence staining was performed after the covalent binding of cells diffused by lactone-digested CFSE with intracellular amines. GBM cells (3–5 × 10^5^) in serum-free medium were stained with CFSE (concentration: 5 μM) at a dilution of 1:1000 and incubated at 37 °C for 20 min in the dark, followed by sedimentation. The sample was then washed with serum-free medium at a ratio of 1:10 to remove free dye liquor. Cells were then seeded into 8-well slides (Millipore, Billerica, MA) and incubated with EVs at different time points and treated under hypoxia condition (1% O_2_) or normoxia condition (21% O_2_). Cells were fixed with 3.7% (w/v) formaldehyde for 5 min at room temperature, observed and imaged under a fluorescence microscope with three fields selected on a random basis.

### ChIP

The EpiQuik Tissue ChIP Kit (48 reactions) (P-2003-2, Epigentek) was used for ChIP. Cells upon reaching 70–80% confluence were fixed with 1% formaldehyde for 10 min to generate the intracellular DNA-protein crosslink, which was then randomly broken by ultra-sonication into fragments (120 w each round of ultra-sonication, 2 s on, 5 s off, 15 cycles in total). Cell fragments were centrifuged at 13,000 × *g* at 4 °C, followed by the division of supernatant into three tubes, respectively, which was separately added with antibody RNA polymerase II (positive control), mouse anti-immunoglobulin G (IgG) (1 mg/mL, provided by ChIP kit) or rabbit anti-IgG (3900, Cell Signaling Technology) (negative control, NC) or antibodies against KDM3A (ab91252, Abcam), H3K27me3 (ab192985, Abcam), CTGF (SimpleChIP® Human CTGF Promoter Primers #14927), H3K27ac (ab4729, Abcam) and H3K4me1 (ab8895, Abcam) for incubation at 4 °C overnight. After IP, de-crosslink was performed and proteins were treated by proteinase K. DNA was eluted and purified using Active Motif’s ChIP DNA purification kit (58002, Millipore). The purified chromatin was quantified by RT-qPCR. The obtained signals from ChIP assay were divided by signals from an input sample. During the assay, 1% of starting chromatin was used as the input, and then a dilution factor of 100 or 6.644 cycles (log2 of 100) was subtracted from the Ct value of diluted input.

### Western blot analysis

Proteins were extracted with protein concentration quantified using BCA method. After separation using 10% sodium dodecyl sulfate-polyacrylamide gel electrophoresis (40 μg proteins each sample), proteins were transferred onto a polyvinylidene fluoride membrane (Millipore), which was blocked by 5% milk powder containing 0.1% Tween-20. Membrane was incubated with primary antibodies including rabbit anti-EZH1 (ab64850, 1:1000, Abcam), mouse anti-KDM3A (ab91252, 1:1000, Abcam), rabbit anti-CTGF (ab6992, 1:1000, Abcam), rabbit anti-iNOS (ab3523, 1:500, Abcam), rabbit anti-Arg-1 (93668, 1:1000, Cell Signaling Technology) and rabbit anti-β-actin (ab179467, 1:5000, Abcam) overnight at 4 °C. Subsequently, the membrane was further incubated with horseradish peroxide-labeled secondary antibody of goat anti-rabbit IgG (ab205718, 1:5000, Abcam) or goat anti-mouse IgG (ab205719, 1:5000, Abcam) for 2 h. Protein bands were developed with enhanced chemiluminescence reagent (BB-3501, Amesham, UK), and image analysis was performed in an imaging system (Bio-Rad, Hercules, CA) with β-actin as a normalizer.

### RNA isolation and quantification

Trizol reagent (Invitrogen, Carlsbad, CA) was used for RNA extraction, and RNA was reversely transcribed into complementary DNA (cDNA) using the MiRcute miRNA first strand cDNA synthesis kit (Tiangen Biotech, Beijing, China) or Primer Script^TM^ One-Step RT-PCR Kit (Takara, Shiga, Japan). RT-qPCR was conducted in ABI 7500 real-time PCR system (Applied Biosystems, Carlsbad, CA) using SYBR Green IReal-time PCR kit (Cowin Bioscience, Beijing, China). Three replicates were set in each well. β-actin was used as an internal reference for mRNA expression. U6 served as an internal reference for intracellular miR-27b-3p expression. Cel-miR-39 (No. miRB00000010-3-1 and MQPS0000071-1-100, Riobio, Guangzhou, China) added during miRNA extraction from EVs was used as an external reference for normalization of difference among EV samples. The relative expression of genes was analyzed by 2-^ΔΔCT^ method. Primers involved in this experiment were mainly designed by primerbank online website, as shown in Table S3.

### Cell counting kit-8 (CCK-8) assay

The proliferative capacity of GBM cells was detected using CCK-8 kit (Dojindo, Kumamoto, Japan). The viable cells were measured at the optimal density (OD) of 450 nm using a microplate (Multiskan Sky Microplate Spectrophoto-meter, Cat. No. 51119570, Thermo Fisher Scientific).

### Flow cytometry for cell proliferation detection

Cell proliferation was evaluated using a Cell Trace CFSE kit (C34554, Thermo Fisher Scientific). The free amino group was labeled by CFSE and dilution after cell division was analyzed by flow cytometry. The cells were seeded in a 6-well plate at 2 × 10^6^ cells/well, cultured for 24 h and incubated with 5 μM CFSE in PBS for 15 min at 37 °C, followed by culture in the medium for 24 h. CFSE fluorescence was analyzed by a FACS Verse flow cytometer (BD Bioscience) with 488 excitation and emission filters. Fluorescence was compared with CFSE-incubated cells at corresponding time points for immediate analysis.

### Transwell assay

The migration and invasion ability of GBM cells was evaluated using Transwell assay by following the manufacturer’s protocol of 24-well plates and 8-mm transwell inserts (Corning Life Science). For migration analysis, GBM cells (5 × 10^4^) suspending in 200 μL serum-free medium were seeded into the apical chambers and macrophages (1 × 10^4^) were seeded into the basolateral chambers supplemented with 800 μL medium containing 10% FBS. For invasion analysis, the insert membranes were coated with Matrigel (50 mL/well, BD Bioscience, Franklin Lakes, NJ), which was allowed to polymerize at 37 °C for 30 min. The basement membrane was hydrated before use and the remaining steps were same as migration analysis. After culture for 24 h at 37 °C, non-migrating or non-invading cells were removed and then stained with 0.1% crystal violet for 30 min. Stained cells (migrated/invaded cells) were counted in five randomly selected fields under inverted light microscope (Carl Zeiss, German).

### Dual-luciferase reporter gene assay

*EZH1* 3’UTR sequences containing binding sites with MUT or WT hsa-miR-27a-3p were constructed by Genscript (Nanjing, China). Both EZH1 3’UTR MUT and WT were cloned into pmirGLO luciferase reporter plasmids (E1330, Promega). HEK293T cells were cultured in 24-well plates for 24 h, which were then co-transfected with pmirGLO-WT-EZH1 or pmirGLO-MUT-EZH1 3’UTR reporter plasmids (0.5 μg), internal reference plasmids and hsa-miR-27a-3p mimic or mimic NC using Lipofectamine 3000 (Invitrogen). After 48 h, Renilla luciferase activity and Firefly luciferase activity were measured by dual luciferase assay system (Promega) with Renilla luciferase activity as internal reference.

### Animal experiment

BALB/c female nude mice (*n* = 60) purchased from SLAC Laboratory Animal (Shanghai, China) were housed in a specific pathogen-free environment with a 12-h light/dark cycle (from 8 a.m. to 8 p.m.), temperature of 23 ± 1 °C, and humidity of 60–70%, and fed with rodent feeding standards. The cages were renewed regularly on a weekly basis, and the mice were given ad libitum access to water using the water bottle. Dedicated persons were responsible for regularly checking various feeding conditions. Each cage had no more than 5 mice to ensure that the mice were in a comfortable state. After 1-week adaptive feeding, the mice were anesthetized with pentobarbital sodium and treated differently with 12 mice for each treatment. Sham-operated mice were taken as the sham group, while others were injected with murine GBM cell line GL261 (with 10^6^ cells/mouse) or macrophage (with 2 × 10^5^ cells/mouse) with adenovirus-mediated CTGF knockdown into the caudate nucleus of the right brain. Subsequently, mice were intravenously injected with PBS or the equivalent volume of EVs (8 mg/kg) extracted from hsa-miR-27a-3p mimic-treated GL261 cells *via* the caudal vein every 3 days. Six mice were randomly selected from each group to record the survival time. The tumors of other mice were dissected 40 days after xenograft, and frozen in liquid nitrogen or fixed in formalin, with serum samples collected.

### ELISA

ELISA kits involving hsa-IL-10 (ab46034, Abcam), mmu-IL-10 (ab46103, Abcam), hsa-TNF-α (ab100654, Abcam) and mmu-TNF-α (ab208348, Abcam) were commercially obtained to detect the levels of IL-10 and TNF-α in the supernatant of U87MG cells, peripheral blood of mice and lysate supernatant of mouse xenograft tumors with 10 μL sample added in each well of a 96-well microtiter plate. The OD value was measured by an automatic microplate reader at wavelength of 450 nm with normalization to diluted antibodies in the medium, ranging from 10 to 2000 pg/mL, and standard curves were plotted.

### H&E staining

Mouse brain tissues were fixed in 10% formalin for 24 h, routinely dehydrated, paraffin-embedded and cut into 3 μm-thick sections. H&E staining was performed following previously described methods [27] to determine the lesion areas in mouse brain tissues. The images were photographed under a microscope.

### Statistical analysis

All data were analyzed using SPSS 21.0 software (IBM Inc., Armonk, NY). Measurement data were expressed by mean ± standard deviation. Unpaired *t*-test was used for comparison between two groups, while one-way analysis of variance (ANOVA) was used for comparison among multiple groups, followed by Tukey’s post hoc test. Comparison among groups at different time points was conducted using two-way ANOVA and Bonferroni’s post hoc test. *p* < 0.05 considered statistically significant.

## Supplementary information

supplementary materials

Table S1

Table S2

WB figures

## Data Availability

The data that supports the findings of this study are available in the manuscript and supplementary materials.
