# Supplementary material for: microRNA-27a-3p delivered by extracellular vesicles from glioblastoma cells induces M2 macrophage polarization via the EZH1/KDM3A/CTGF axis
Source: Cell Death Discov. 2022 May 14;8:260. doi: 10.1038/s41420-022-01035-z (PMC9107457; doi:10.1038/s41420-022-01035-z)
Supplement: Supplementary file 1 — supplementary materials [file 41420_2022_1035_MOESM1_ESM.pdf]

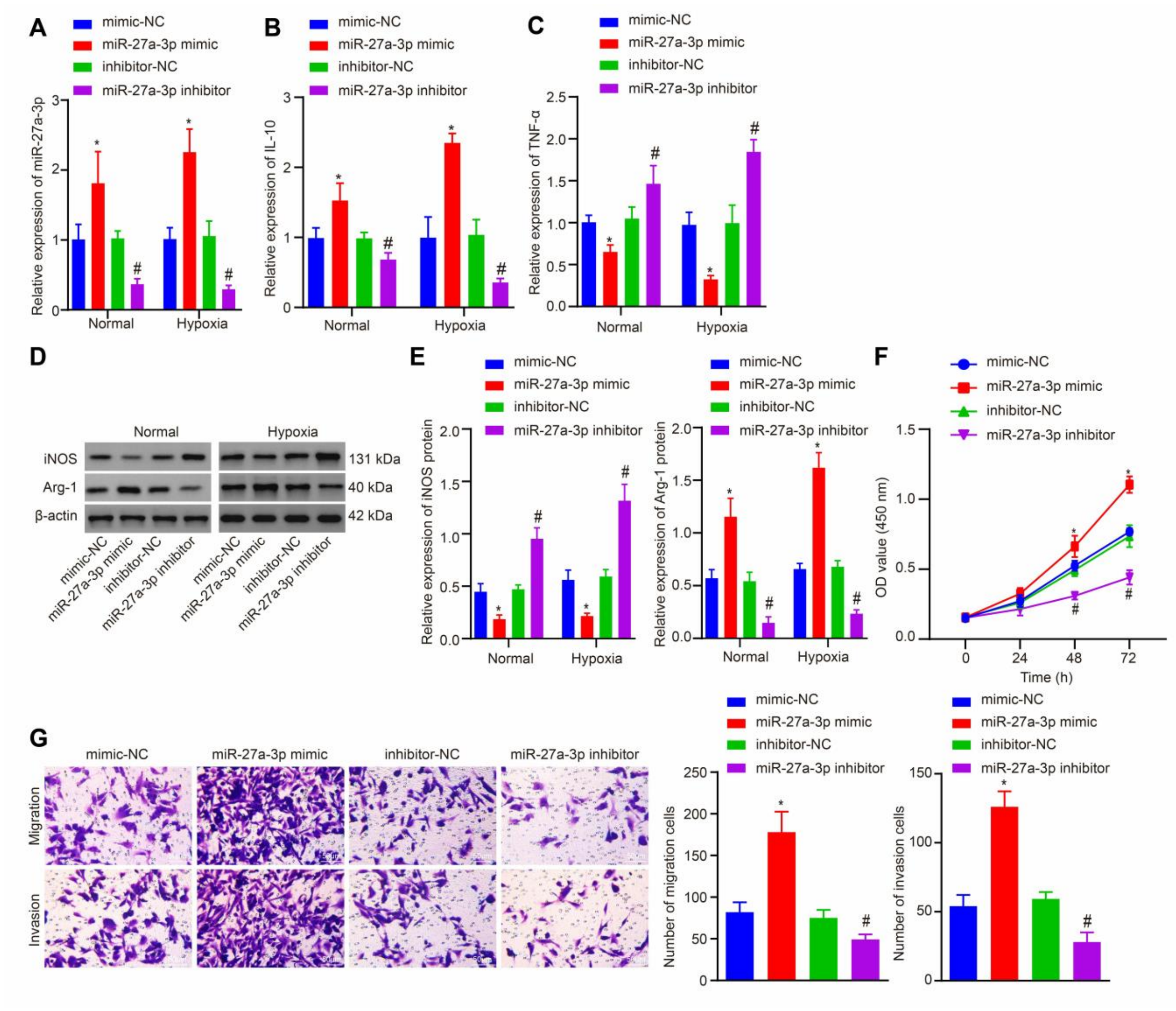

Fig. S1 hsa-miR-27a-3p contributes to M2 macrophage polarization. A, Expression of hsa-miR-27a-3p in macrophages treated with hsa-miR-27a-3p mimic/inhibitor under normoxia/hypoxia conditions determined by RT-qPCR. B, mRNA expression of *IL-10* in macrophages treated with hsa-miR-27a-3p mimic/inhibitor under normoxia/hypoxia conditions determined by RT-qPCR. C, mRNA expression of *TNF-α* in macrophages treated with hsa-miR-27a-3p mimic/inhibitor under normoxia/hypoxia conditions determined by RT-qPCR. D, Western blot analysis results of the protein expression of iNOS in macrophages treated with hsa-miR-27a-3p mimic/inhibitor under normoxia/hypoxia conditions. E, Western blot analysis results of the protein expression of Arg-1 in macrophages treated with hsa-miR-27a-3p mimic/inhibitor under normoxia/hypoxia conditions. F, The proliferative ability of GBM cells co-cultured with macrophages treated with hsa-miR-27a-3p

mimic/inhibitor under normoxia/hypoxia conditions detected by CCK-8 assay. G, Transwell assay results of migration and invasion ability of GBM cells co-cultured with macrophages treated with hsa-miR-27a-3p mimic/inhibitor under normoxia/hypoxia conditions (scale bar = 50 μm). * $p < 0.05$ compared with macrophages treated with mimic-NC. # $p < 0.05$ compared with macrophages treated with inhibitor-NC. The experiment was repeated 3 times independently.

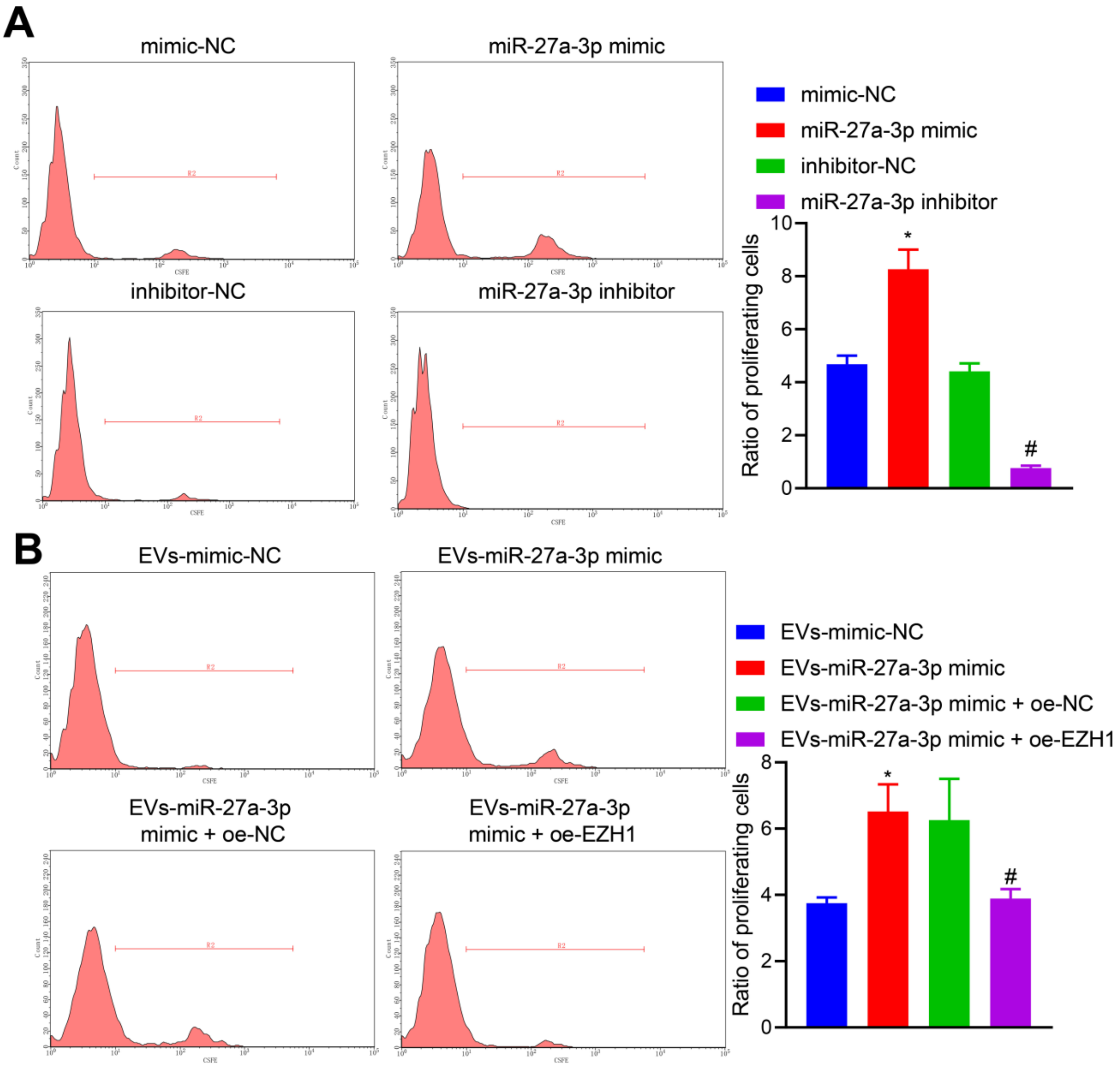

Fig. S2 GBM cell proliferation analyzed by flow cytometry. A, The proliferative ability of GBM cells co-cultured with macrophages treated with hsa-miR-27a-3p mimic/inhibitor. B, The proliferative ability of GBM cells co-cultured with macrophages treated with hsa-miR-27a-3p mimic in presence/absence of oe-EZH1 following co-culture with GBM-EVs. * $p < 0.05$ compared with the mimic-NC or EVs-mimic-NC group. # $p < 0.05$ compared with the inhibitor-NC or EVs-hsa-miR-27a-3p mimic + oe-NC group. The experiment was repeated 3 times independently.

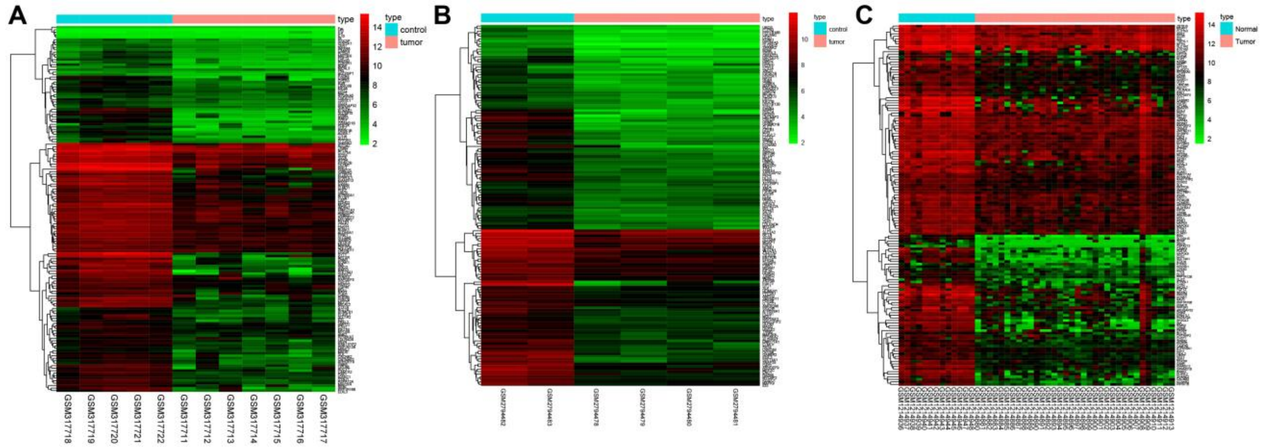

Fig. S3 A, Heatmap of differentially expressed genes in GSE12657. B, Heatmap of differentially expressed genes in GSE104291. C, Heatmap of differentially expressed genes in GSE50161. The abscissa is the sample number, the ordinate is the gene name, and the histogram on the upper right is the color level.

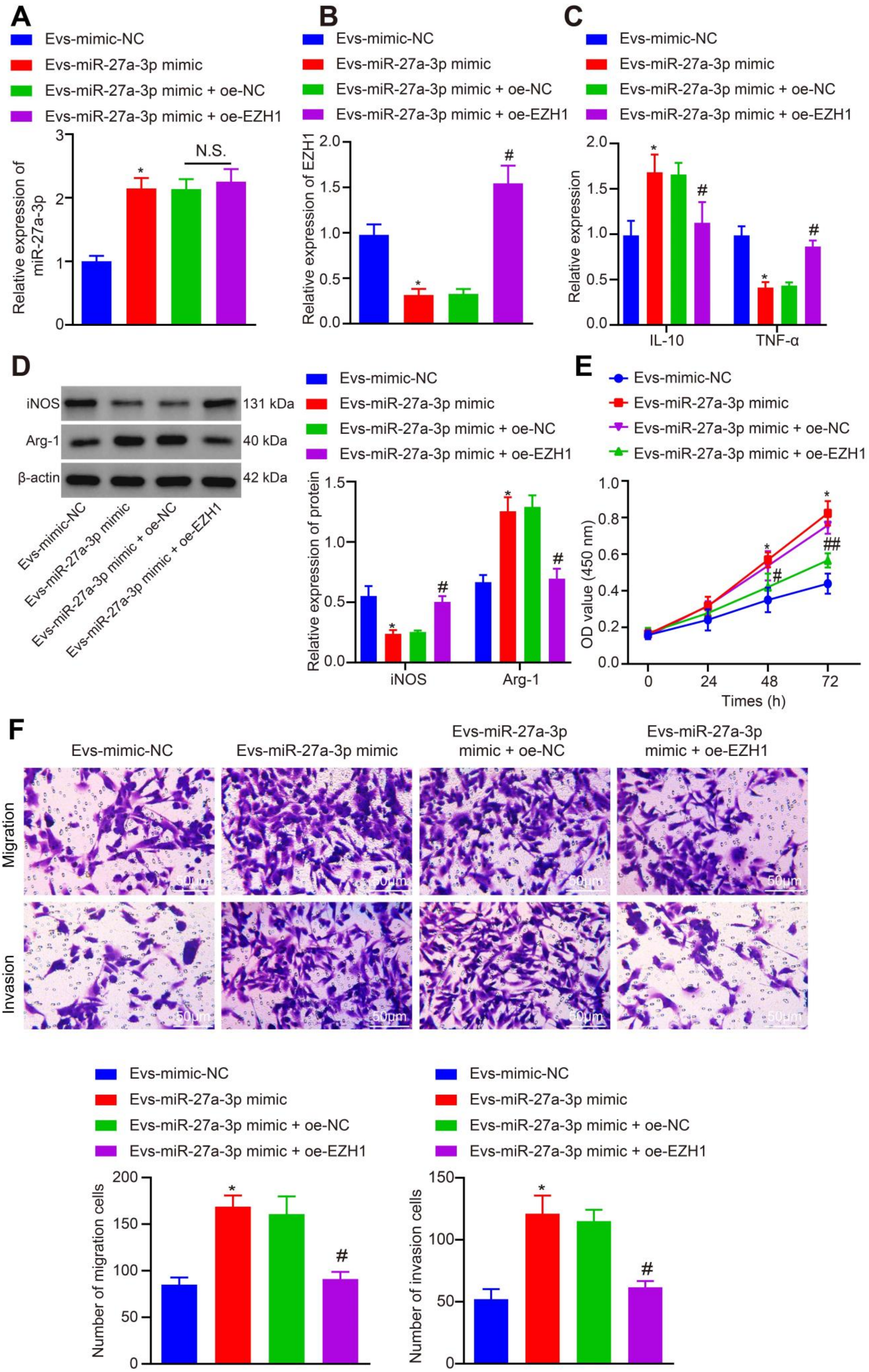

A
B
C
D
E
F
Evs-mimic-NC
Evs-miR-27a-3p mimic
Evs-miR-27a-3p mimic + oe-NC
Evs-miR-27a-3p mimic + oe-EZH1
Relative expression of miR-27a-3p
N.S.
Relative expression of EZH1
Relative expression
IL-10
TNF-α
iNOS
Arg-1
β-actin
131 kDa
40 kDa
42 kDa
Relative expression of protein
OD value (450 nm)
Times (h)
Migration
Invasion
50μm
Number of migration cells
Number of invasion cells

Fig. S4 GBM-EV-derived hsa-miR-27a-3p downregulates *EZH1* to induce M2 macrophage polarization, facilitating GBM cell proliferation, migration and invasion. A, The expression of hsa-miR-27a-3p in macrophages cultured under hypoxic conditions determined by RT-qPCR. B, mRNA expression of *EZH1* in macrophages cultured under hypoxic conditions determined by RT-qPCR. C, mRNA expression of *IL-10* and *TNF-α* in macrophages cultured under hypoxic conditions determined by RT-qPCR. D, The protein expression of iNOS and Arg-1 determined by Western blot analysis. E, The proliferation ability of GBM cells evaluated by CCK-8 assay. F, The migration and invasion ability of GBM cells assessed by Transwell assay (scale bar = 50 μm). * $p < 0.05$ compared with the EVs-mimic-NC group. # $p < 0.05$ compared with the EVs-miR-27a-3p mimic + oe-NC group. The experiment was repeated 3 times independently.

Table S3 Primer sequences for RT-qPCR

	Human	Mouse
miR-27a-3p	F 5-GCGCGTTCACAGTGGCTAAG-3	
	R universal primer	
U6	F 5-AGAGAAGATTAGCATGGCCCCTG-3	
	R universal primer	
EZH1	F 5-GCTGTTGTGTCCTGCCATTC-3	F 5-CCGCTGCATTCCATGAGGA-3
	R 5-AAGGGTGAAGGAAGCAGTCG-3	R 5-TTCTTGTTACCGGCAGGTCC-3
KDM3A	F 5-GAGAGGGGAGAGAAAGGGAGGAG-3	F 5-GTCAGAGCTAGAGTCGGCTG-3
	R 5-TGTACGTTATTGCAGGCCACT-3	R 5-CCCACCAATACTGGCCAACT-3
CTGF	F 5-CACCCGGGTTACCAATGACA-3	F 5-AGCGGTGAGTCCTTCCAAAG-3
	R 5-TCCGGGACAGTTGTAATGGC-3	R 5-TTCATGATCTCGCCATCGGG-3
IL-10	F 5-AGACAGACTTGCAAAAGAAGGC-3	F 5-GCTCCAAGACCAAGGTGTCT-3
	R 5-TCGAAGCATGTTAGGCAGGTT-3	R 5-AGGACACCATAGCAAAGGGC-3
TNF-α	F 5-CTGGGCAGGTCTACTTTGGG-3	F 5-ACCCTCACACTCACAAACCA-3
	R 5-CTGGAGGCCCCAGTTTGAAT-3	R 5-ACCCTGAGCCATAATCCCCT-3
β-actin	F 5-ATCGTGCGTGACATTAAGGAGAAG-3	F 5-GCAGGAGTACGATGAGTCCG-3
	R 5-AGGAAGGAAGGCTGGAAGAGTG-3	R 5-ACGCAGCTCAGTAACAGTCC-3

Notes: F, Forward; R, Reverse; miR, microRNA; EZH1, enhancer of zeste 1; KDM3A, lysine demethylase 3A; CTGF, connective tissue growth factor; IL-10, interleukin-10; TNF, tumor necrosis factor; RT-qPCR, reverse transcription quantitative polymerase chain reaction
