# Supplementary material for: microRNA-27a-3p delivered by extracellular vesicles from glioblastoma cells induces M2 macrophage polarization via the EZH1/KDM3A/CTGF axis
Source: Cell Death Discov. 2022 May 14;8:260. doi: 10.1038/s41420-022-01035-z (PMC9107457; doi:10.1038/s41420-022-01035-z)
Supplement: Supplementary file 4 — WB figures [file 41420_2022_1035_MOESM4_ESM.docx]

Figure 1C:


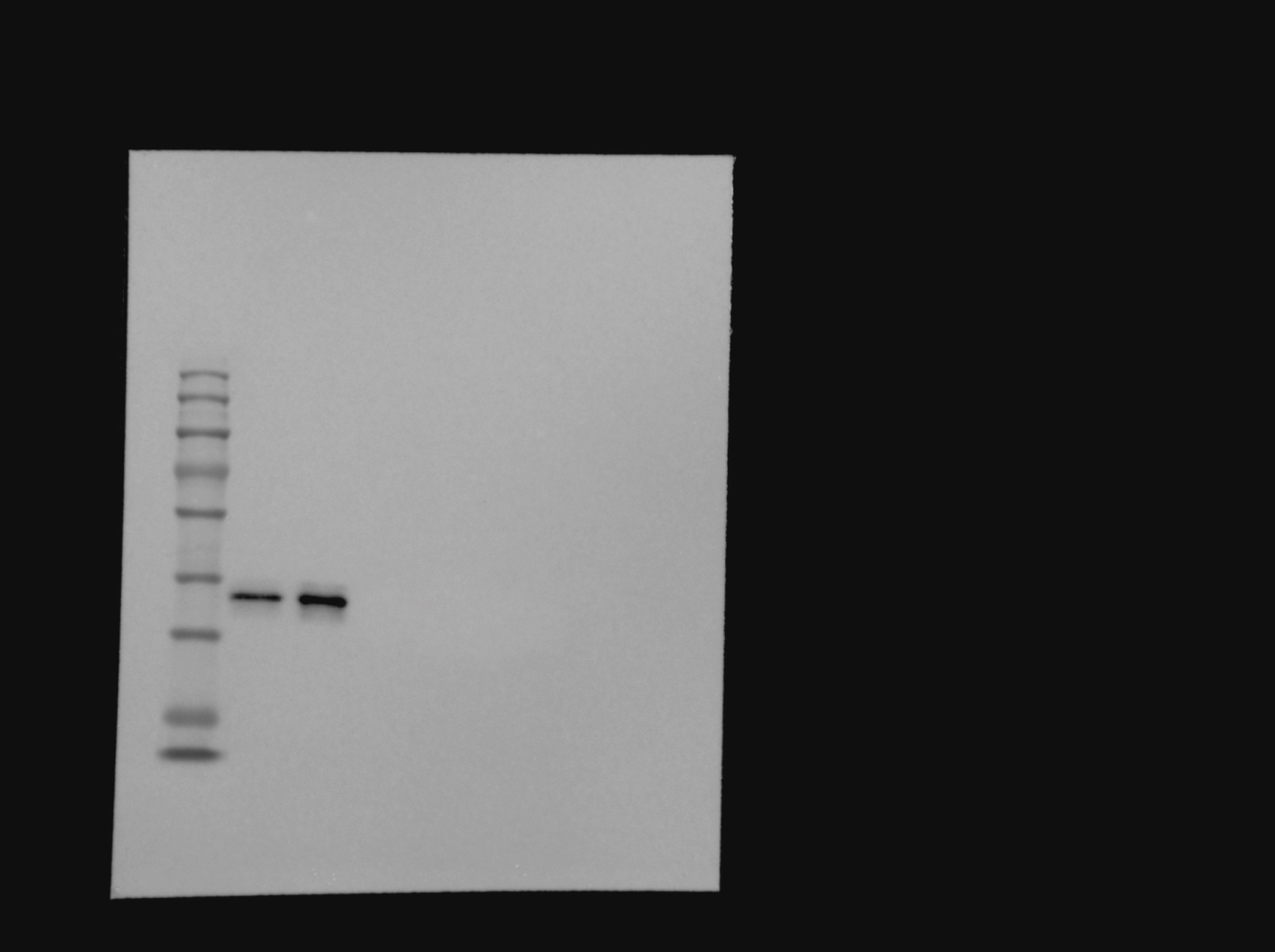
 C-1


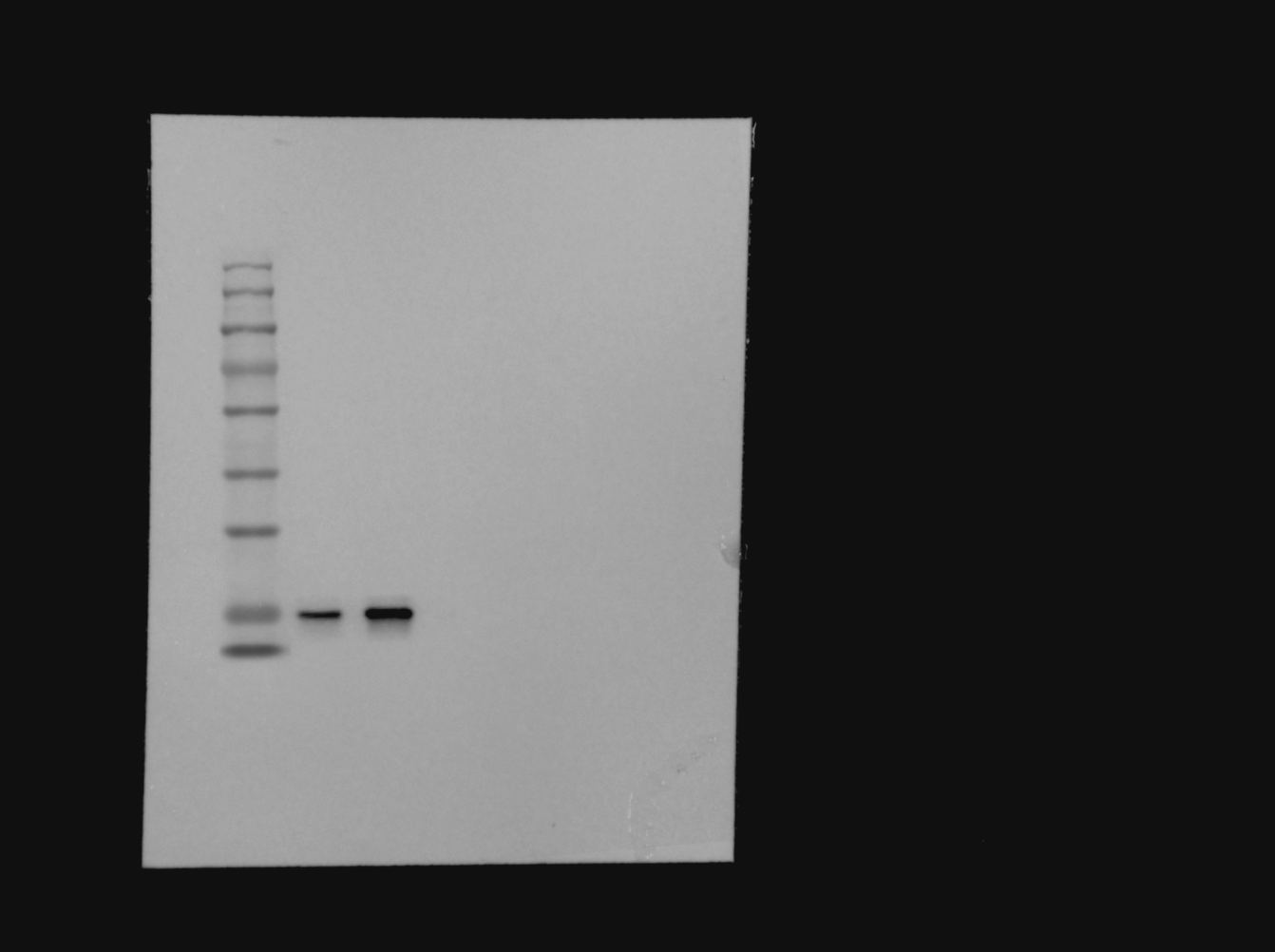
 C-2


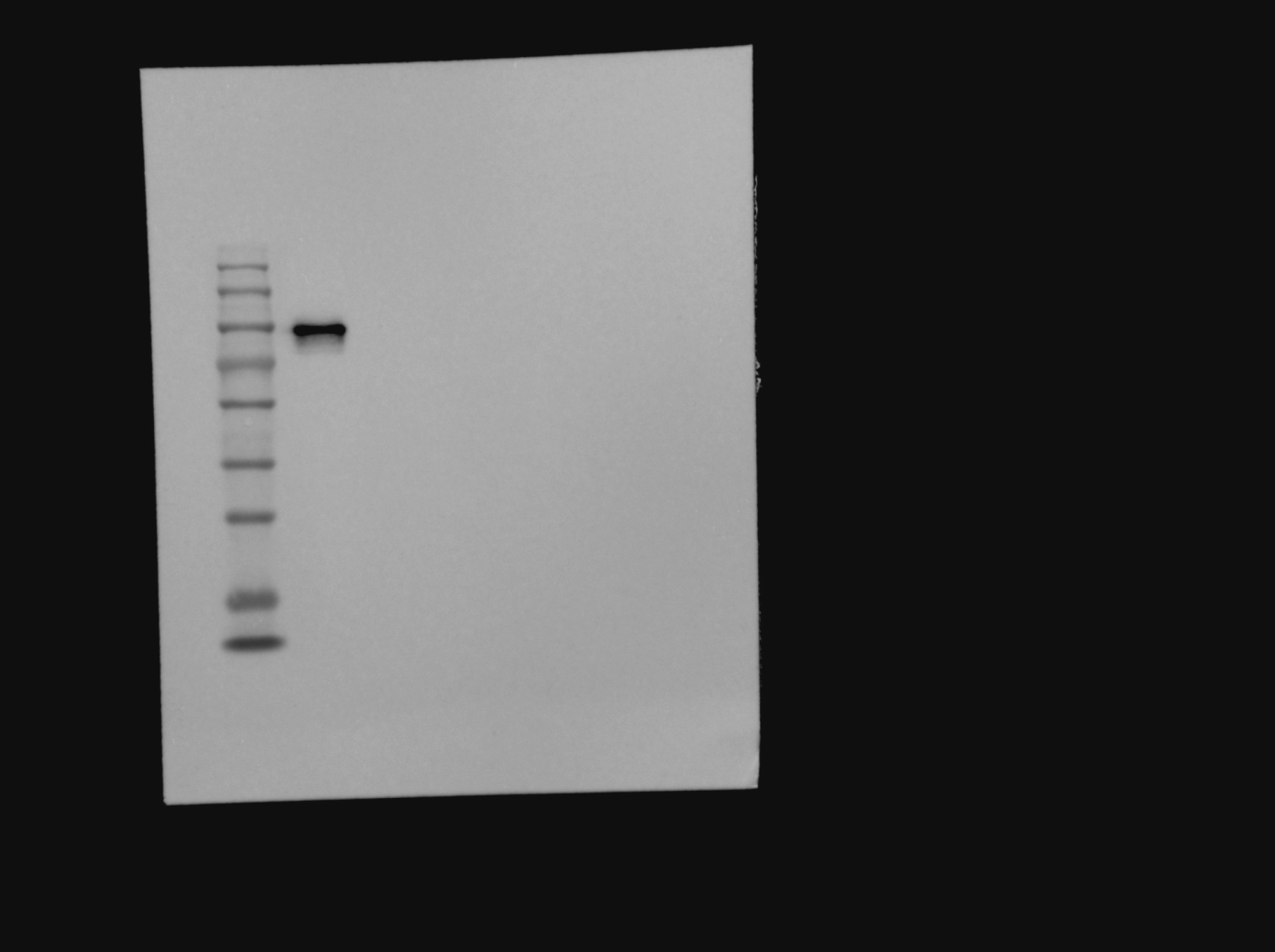
 C-3

Figure 4G:


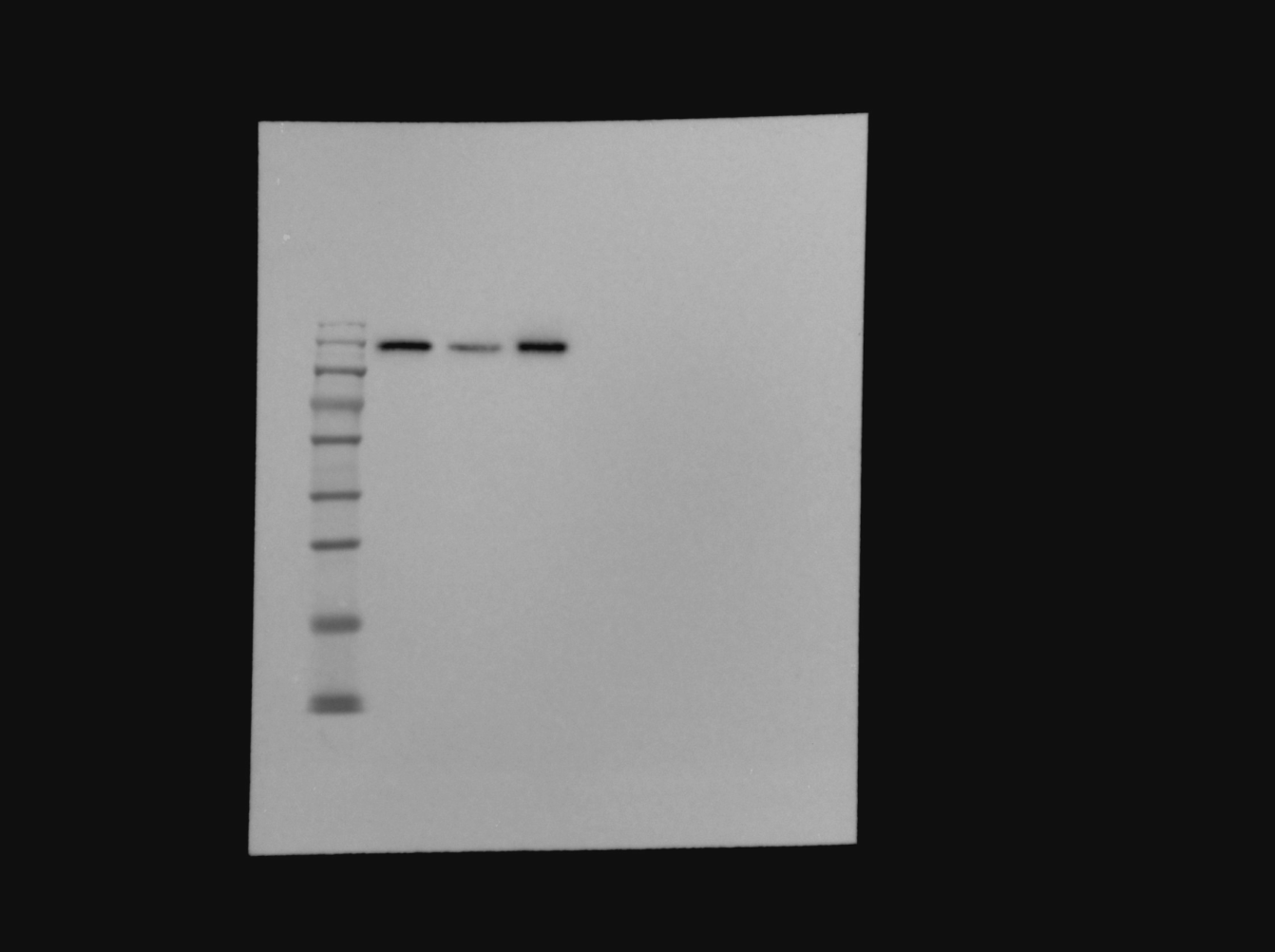
 G-1


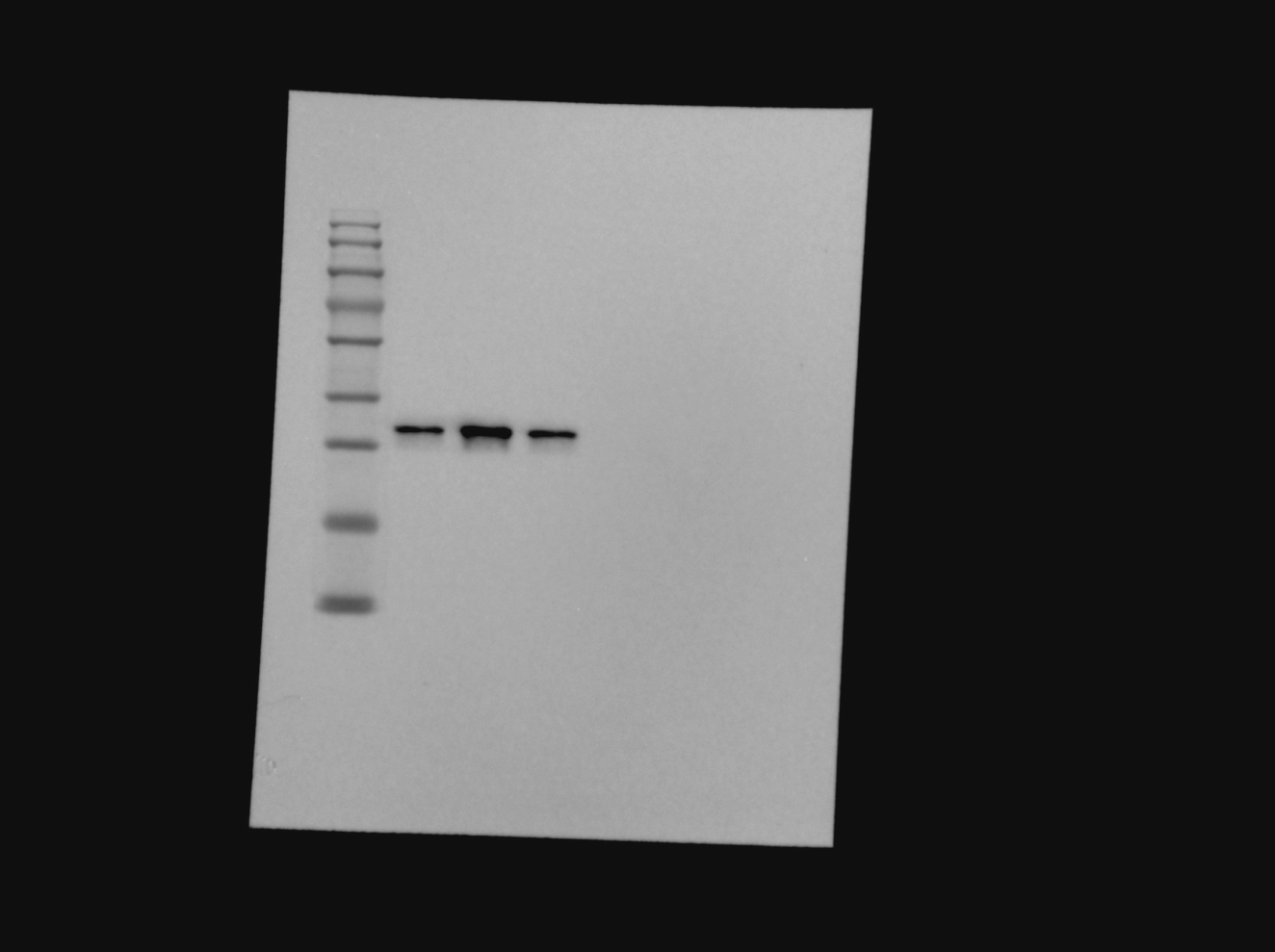
 G-2


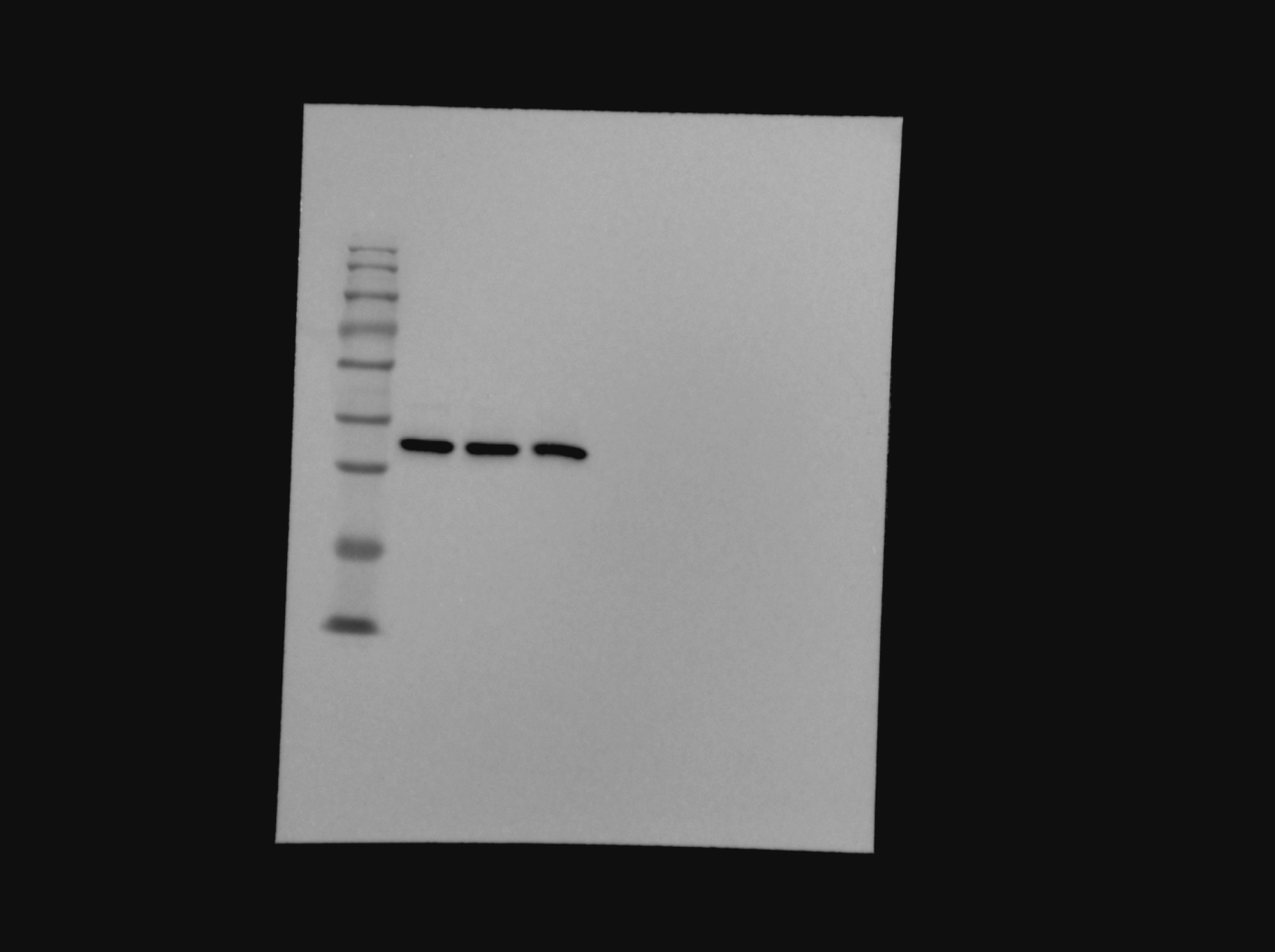
 G-3

Figure 5F:


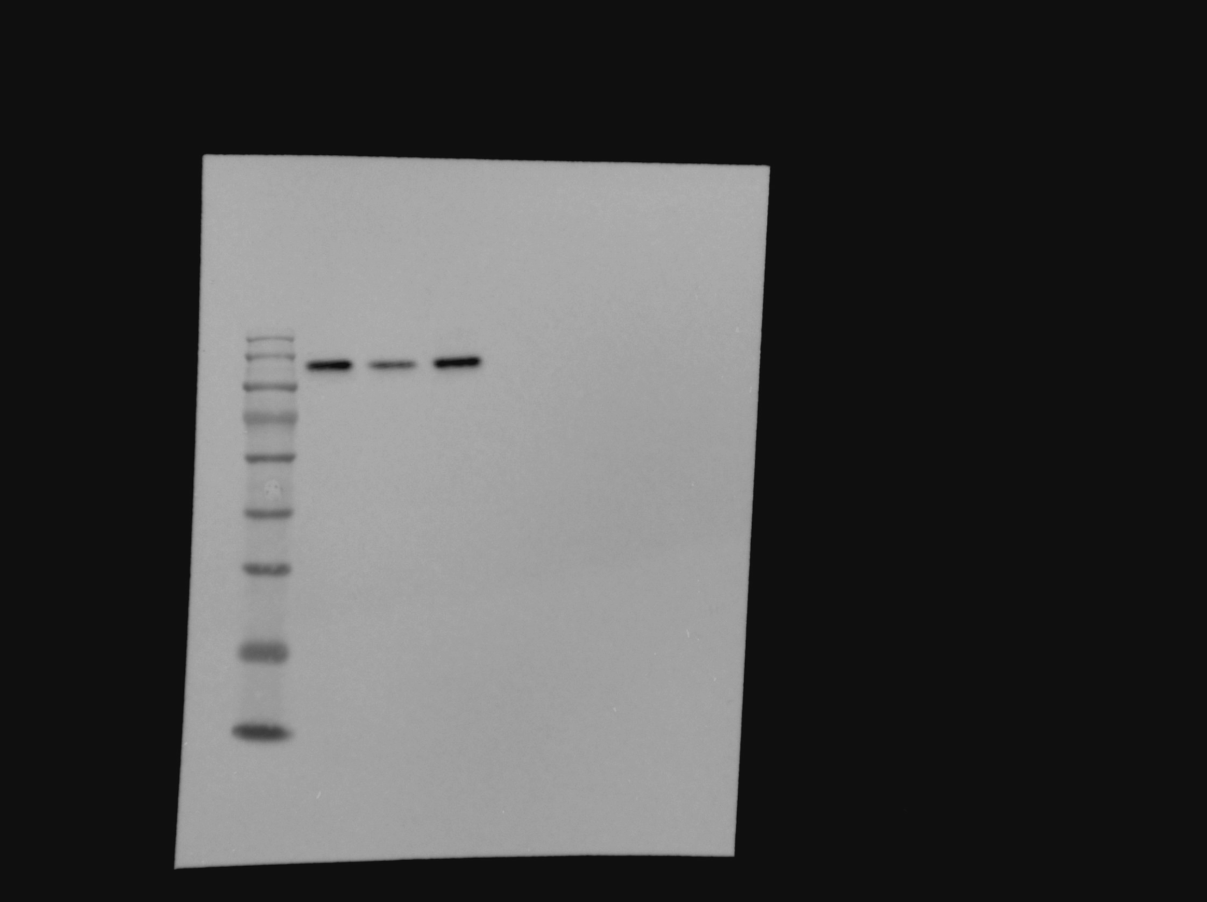
 F-1


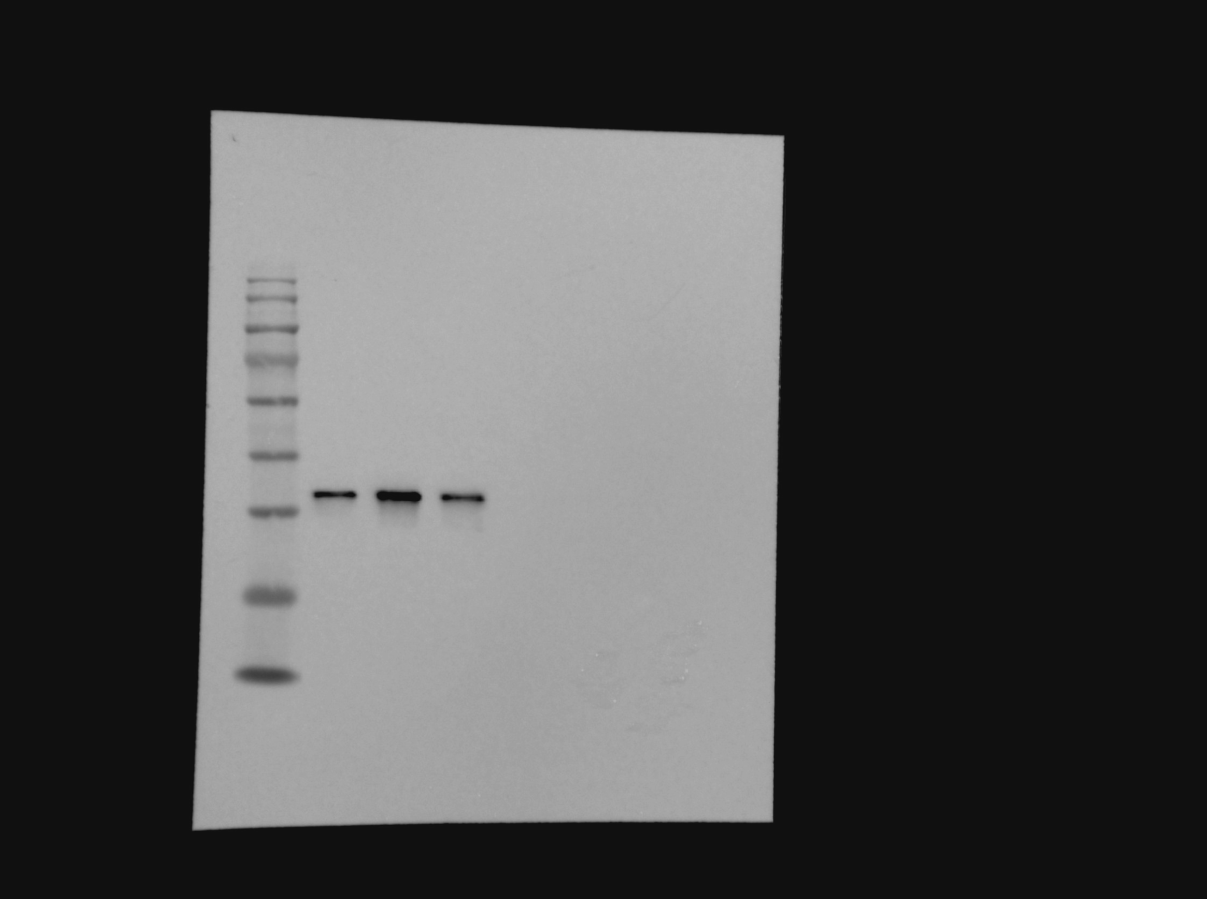
 F-2


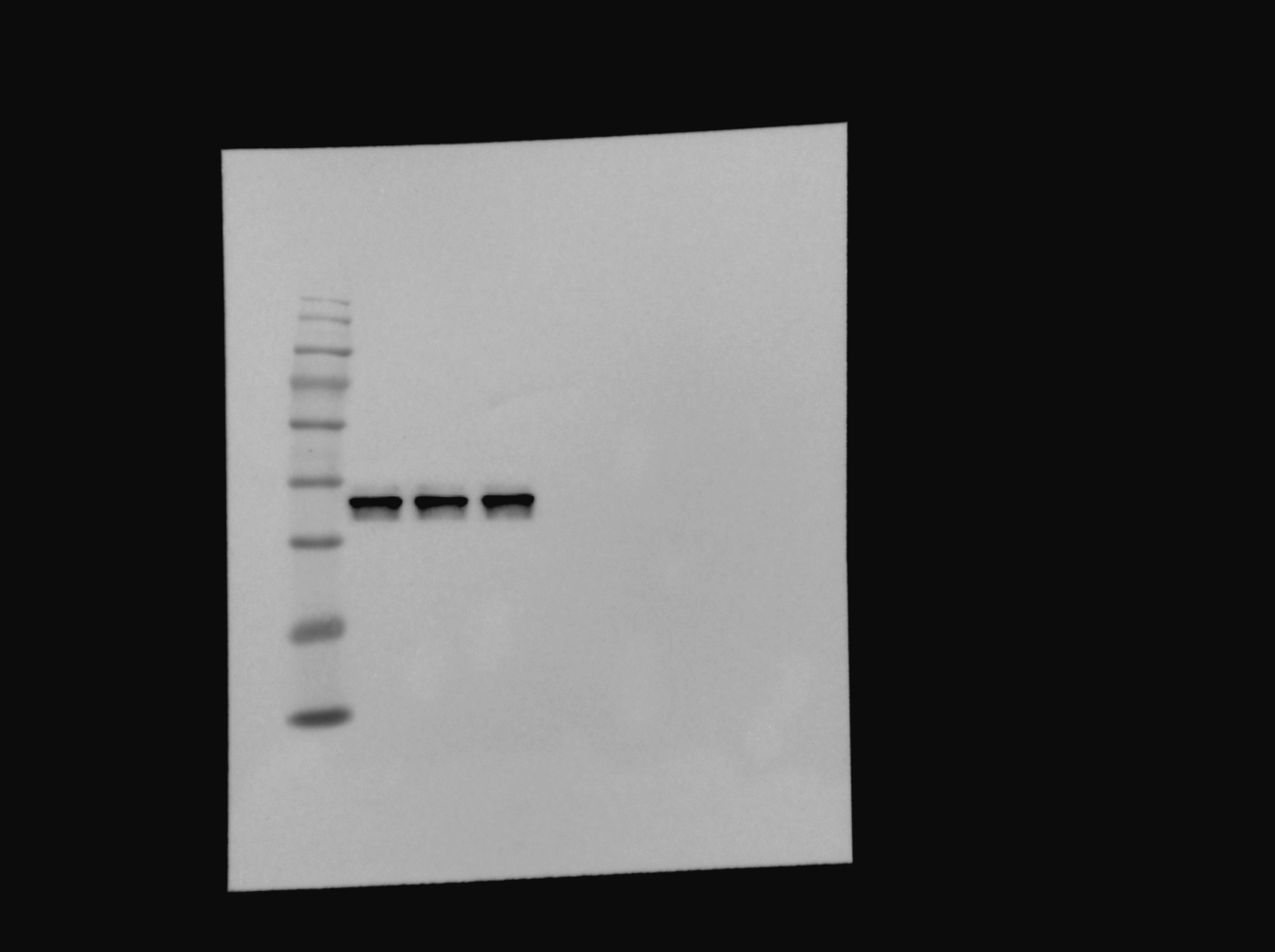
 F-3

Figure 6K:


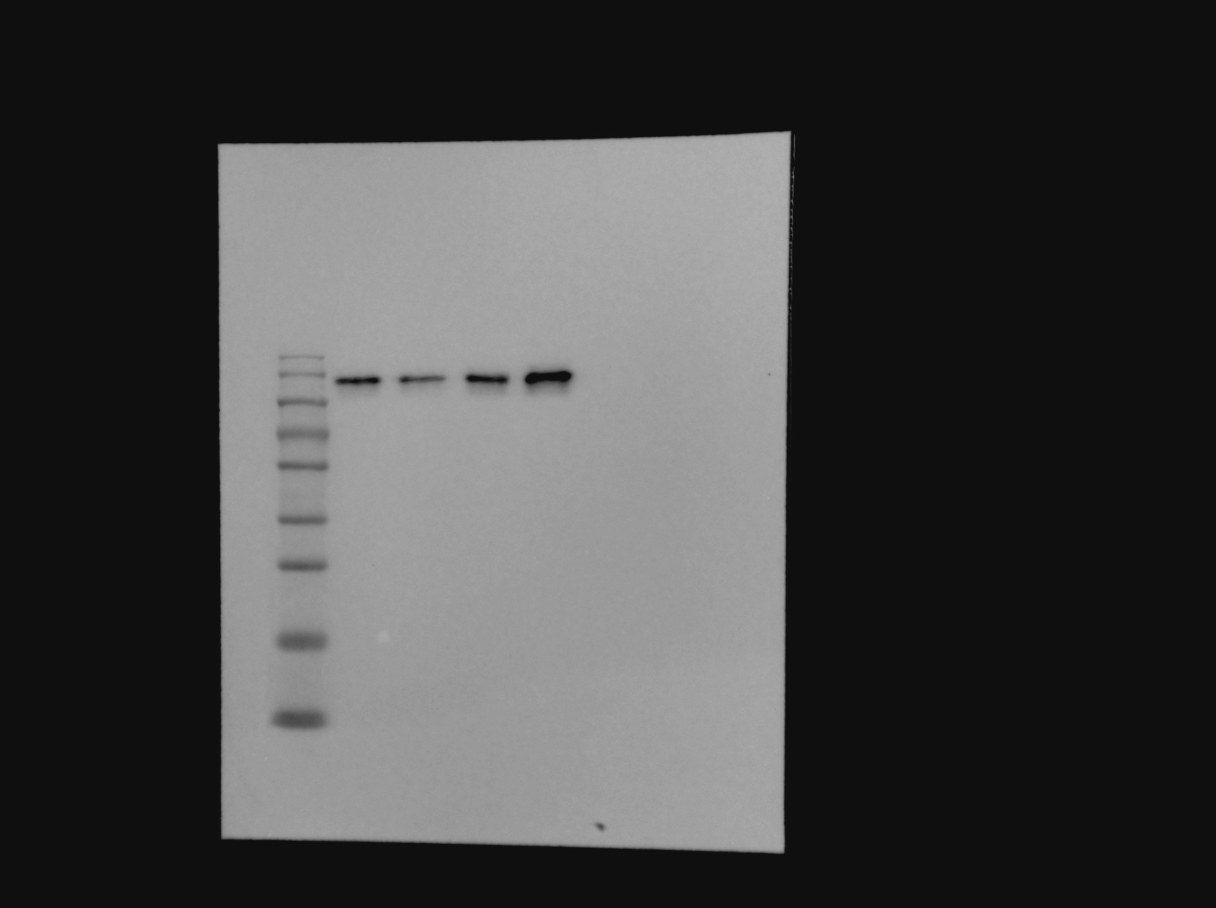
 K-1


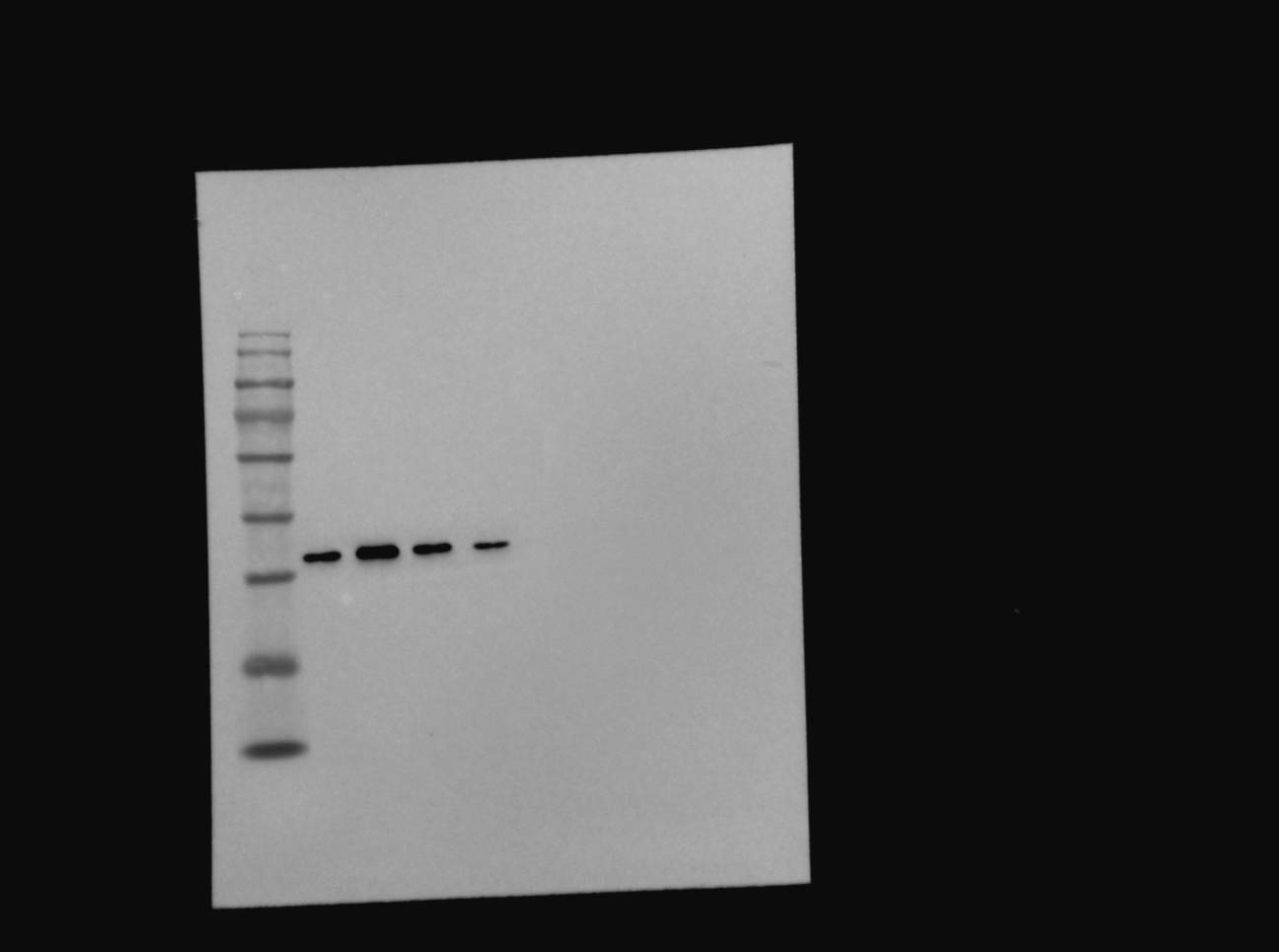
 K-2


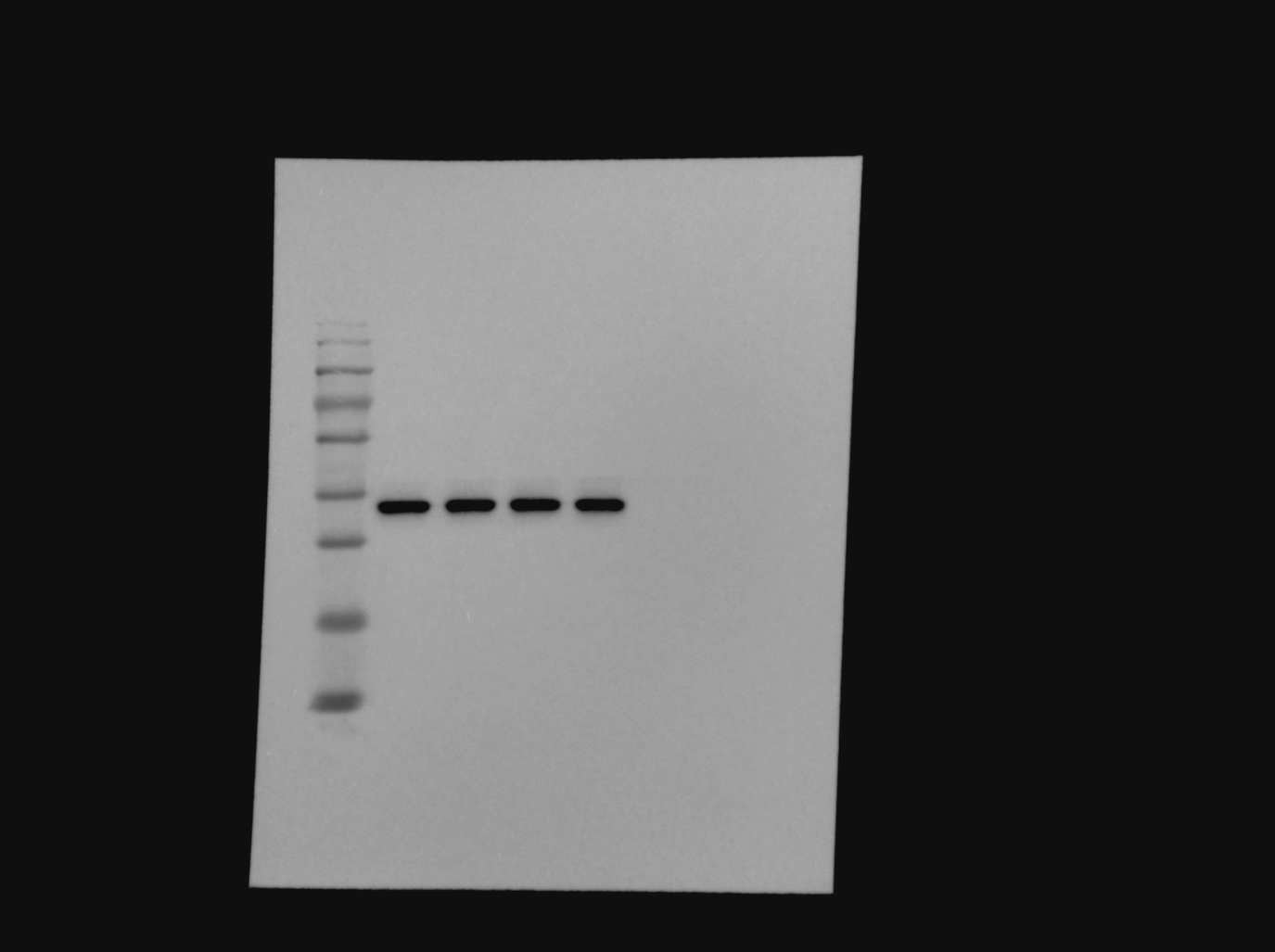
 K-3

Figure 7D:


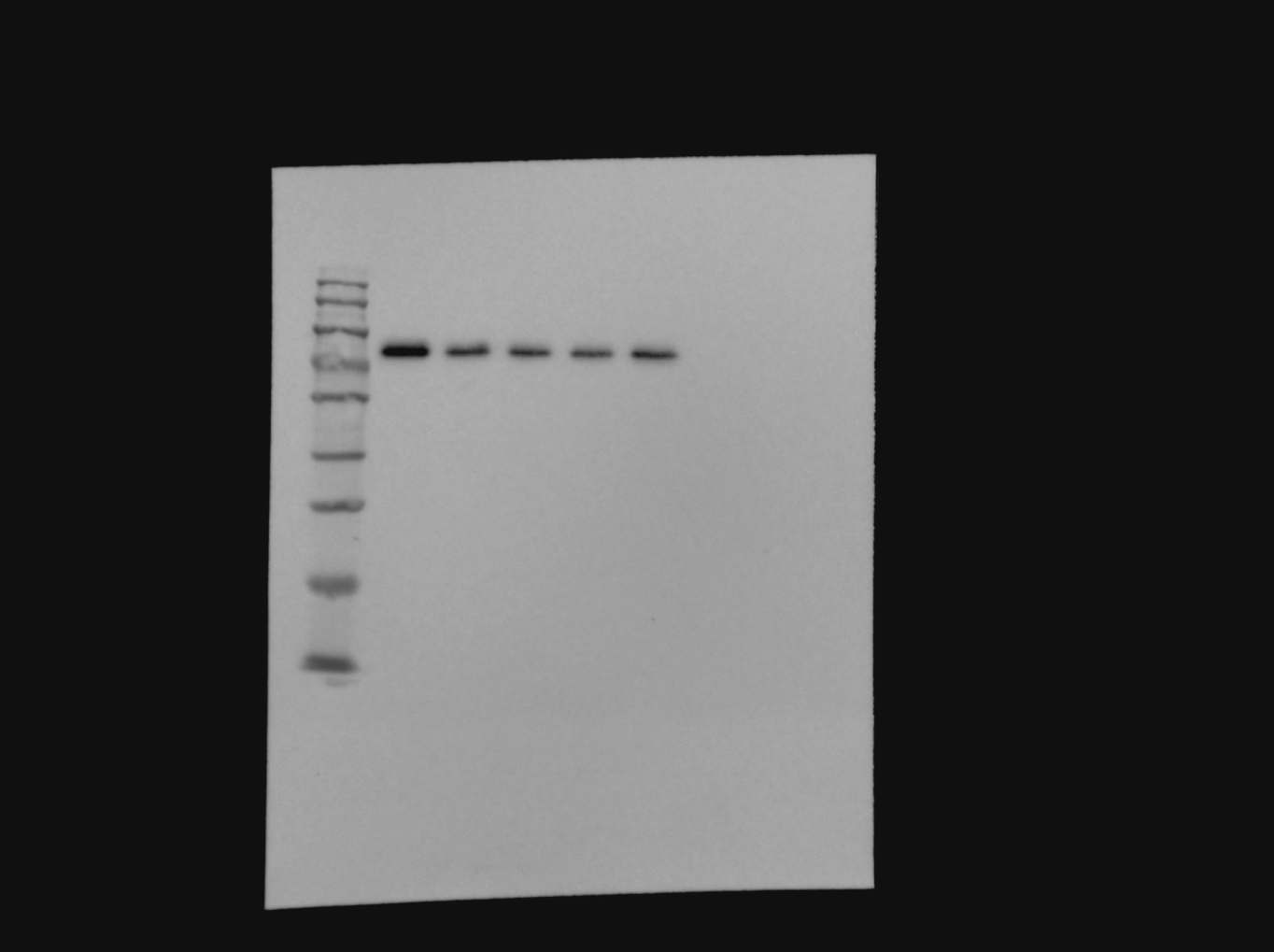
 D-1


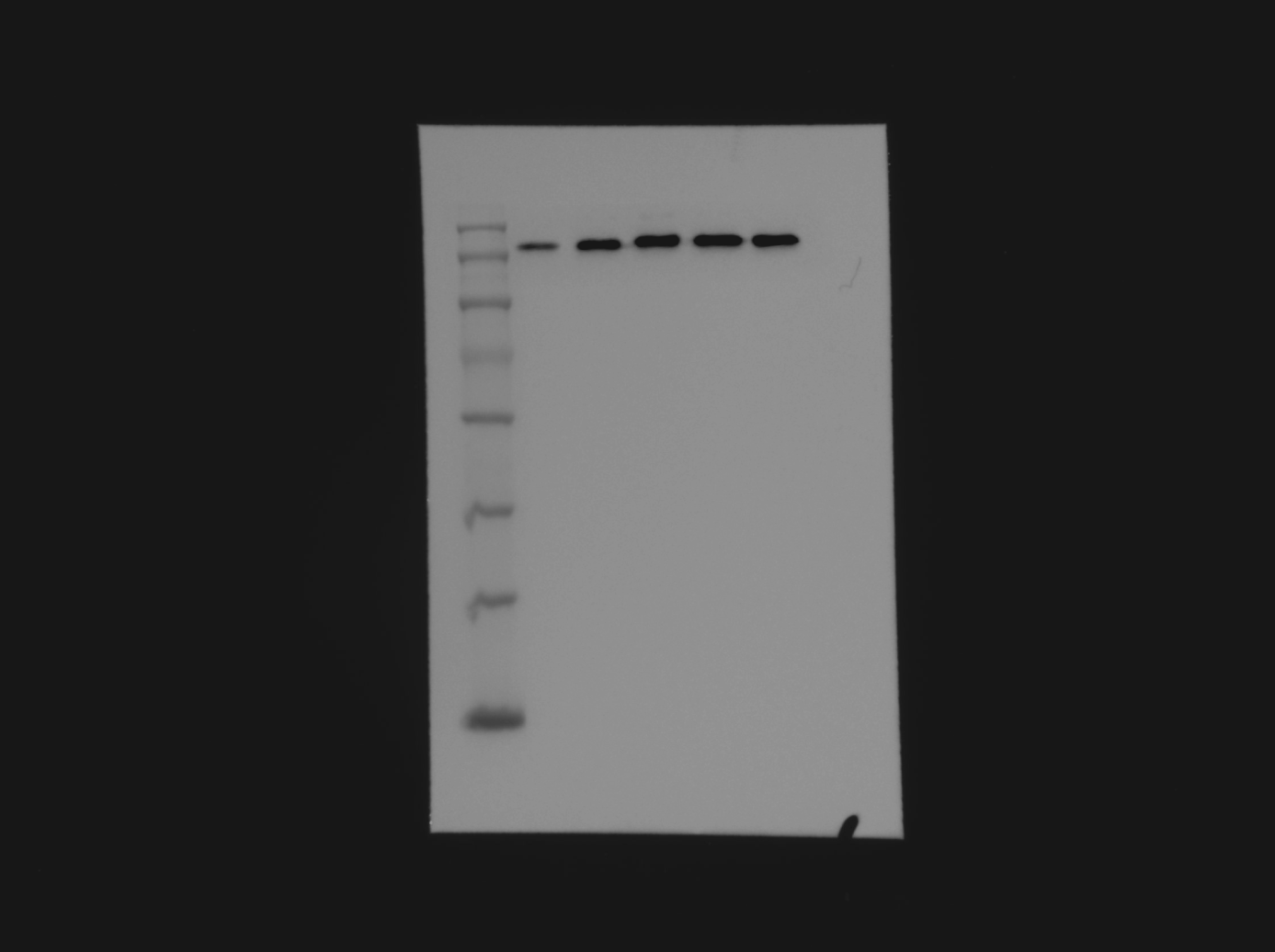
 D-2


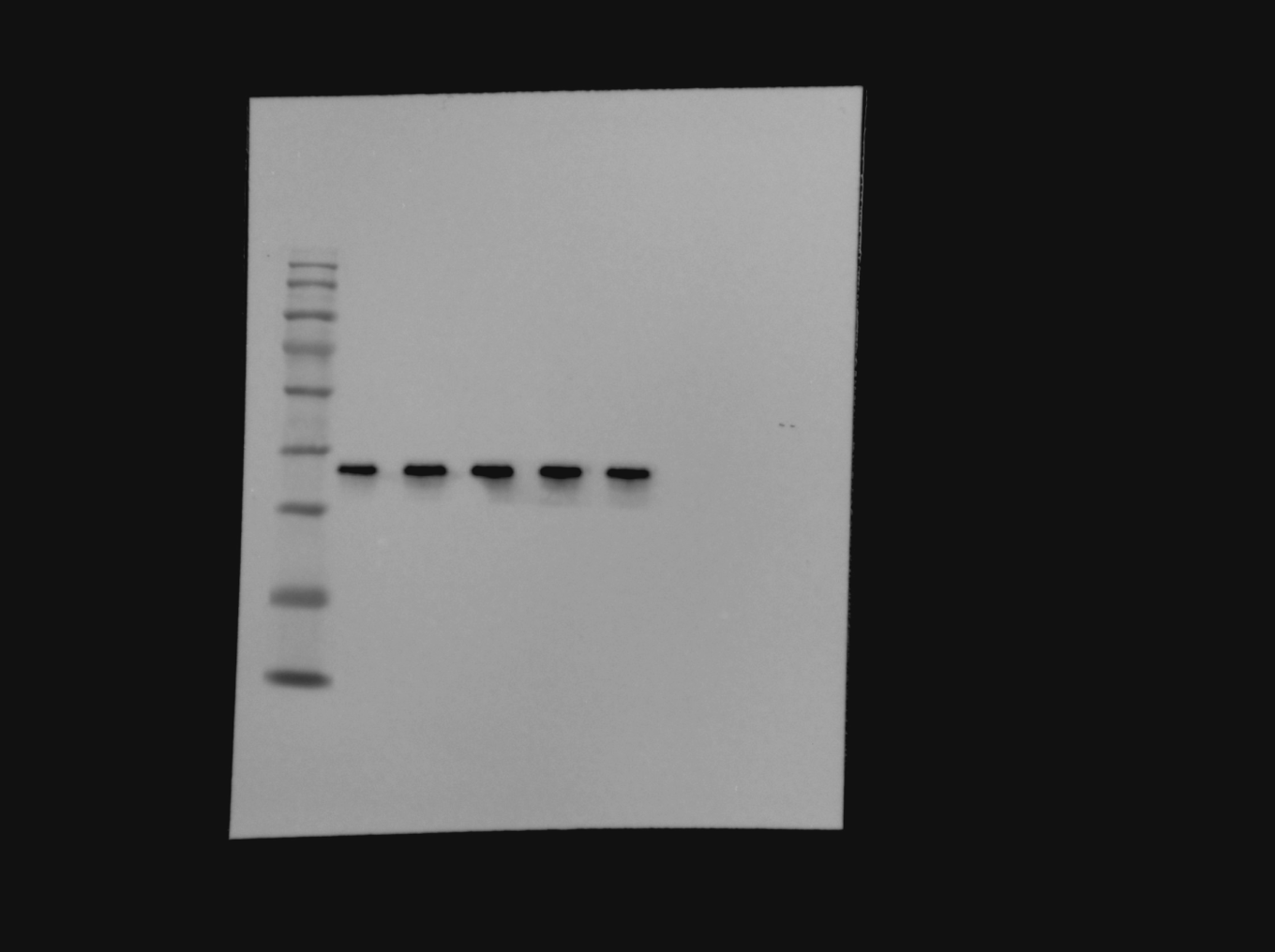
 D-3


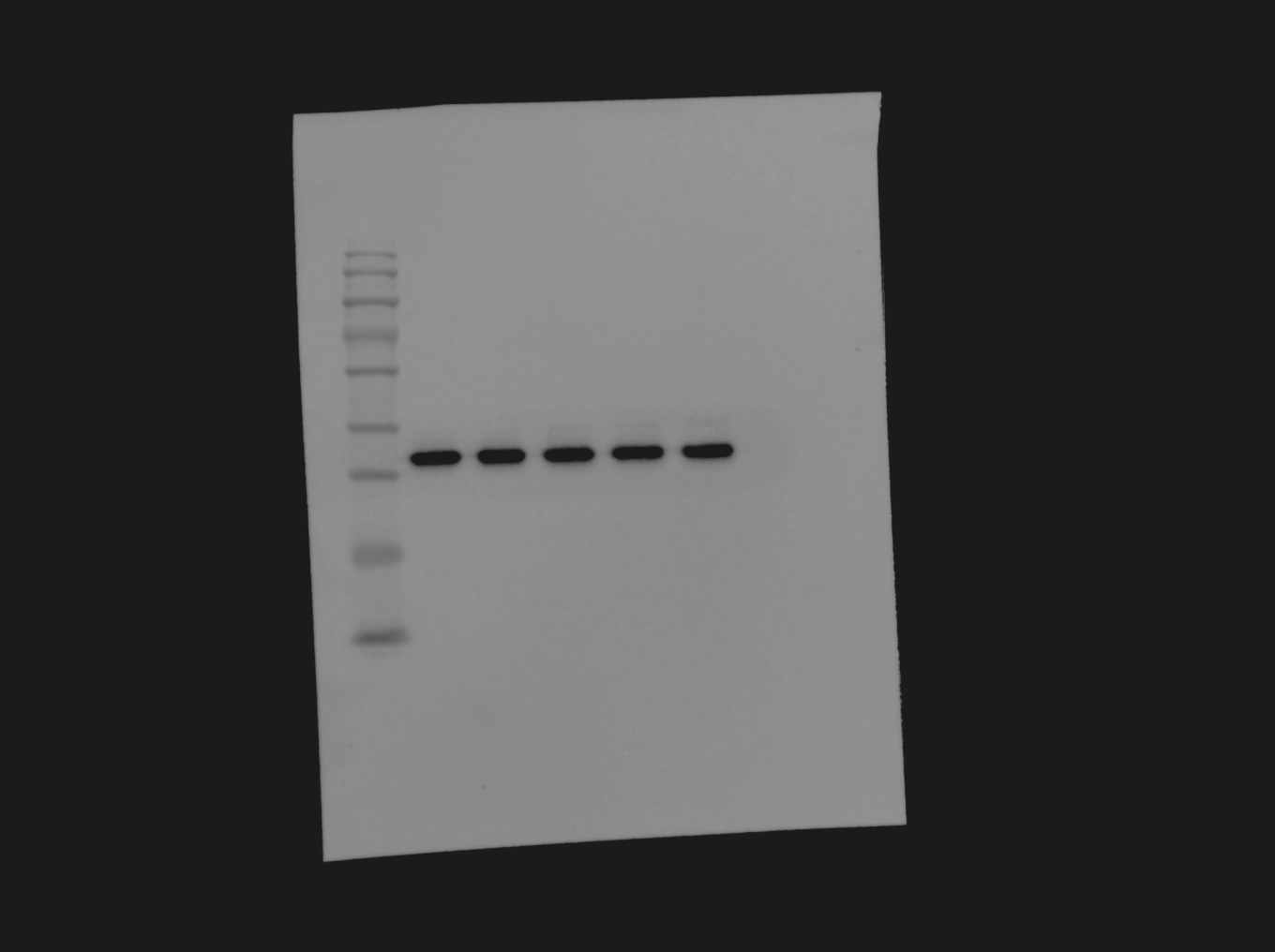
 D-4

Figure S1D:


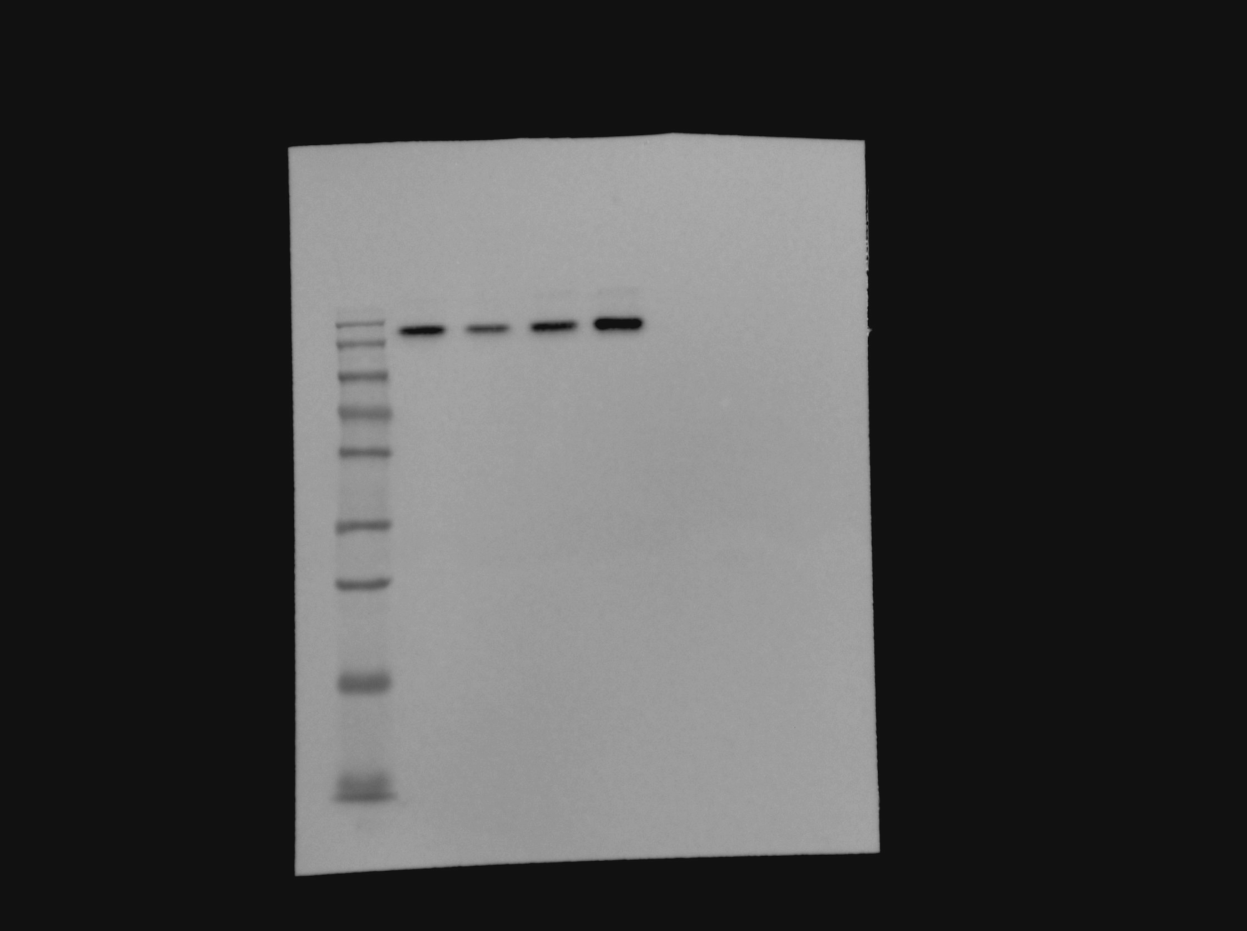
 S1D-1


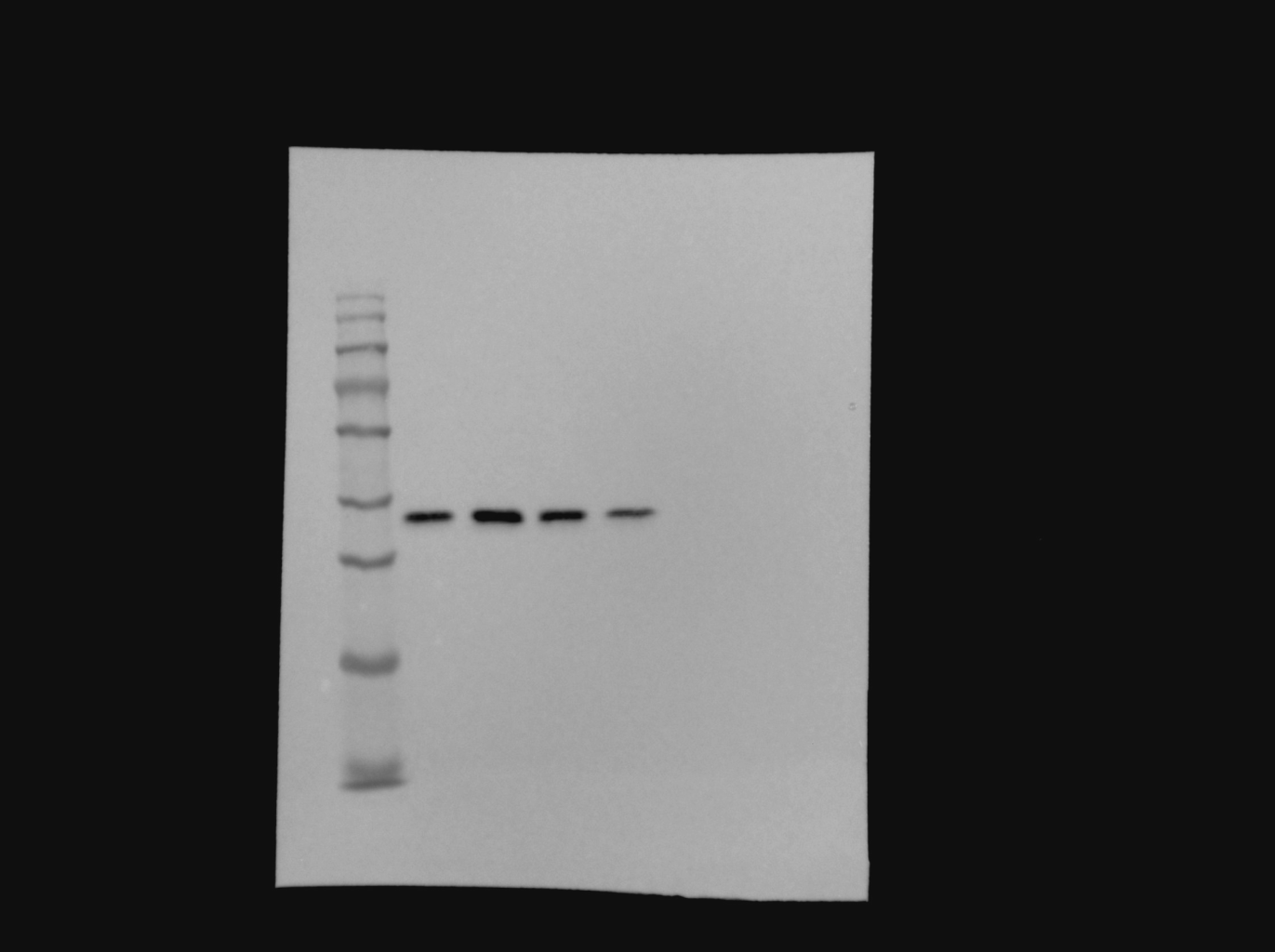
 S1D-2


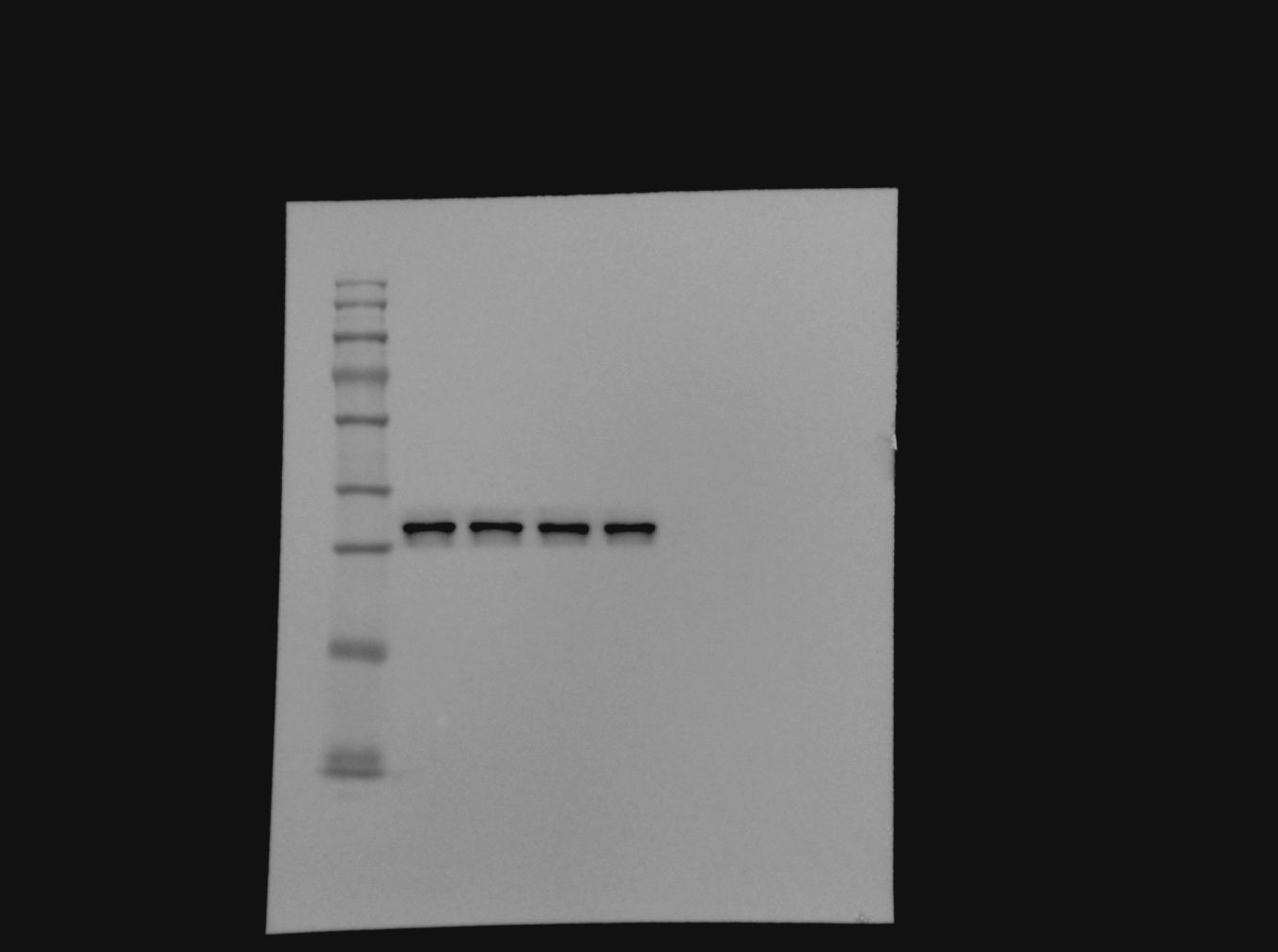
 S1D-3


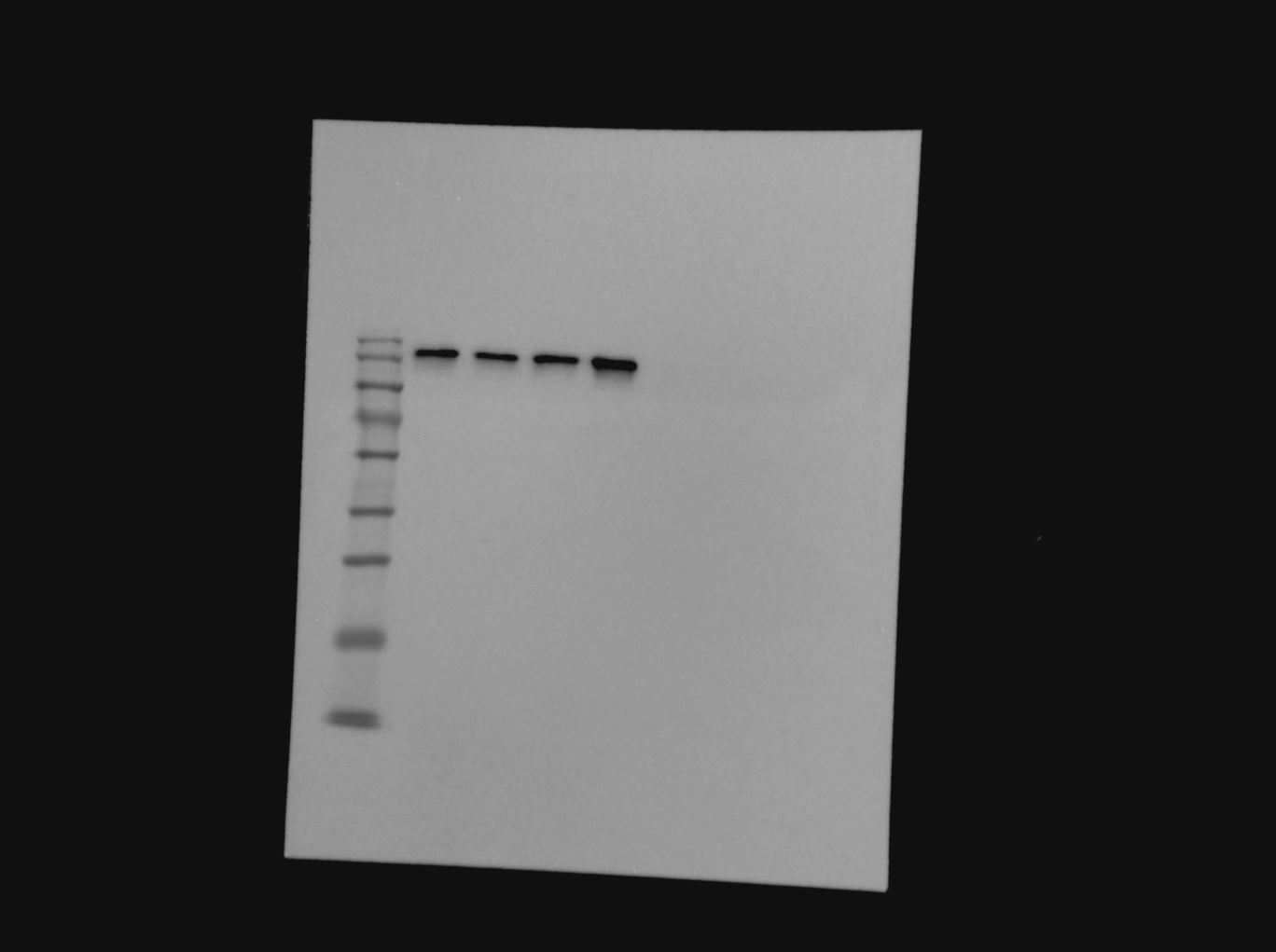
 S1D-4


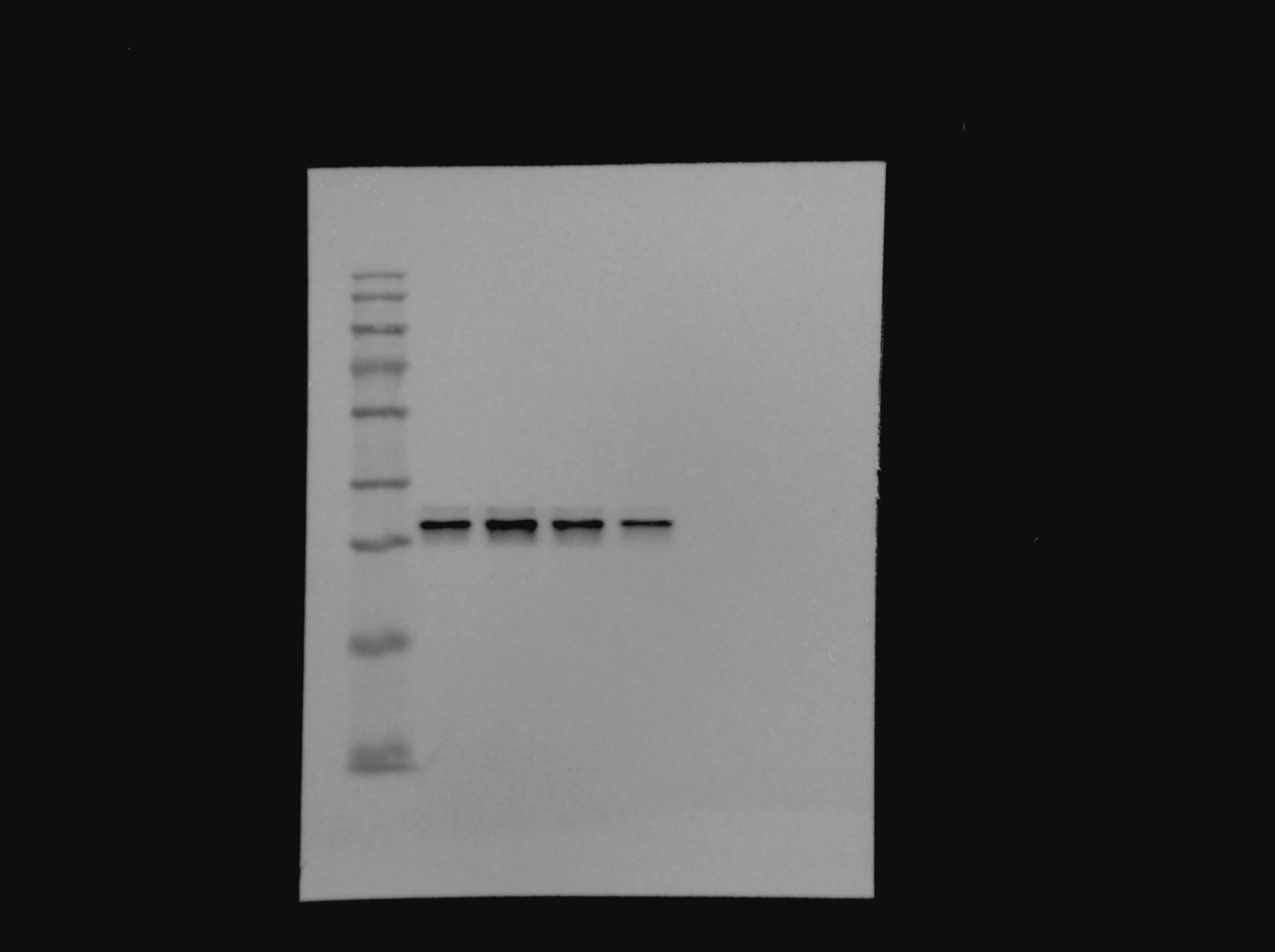
 S1D-5


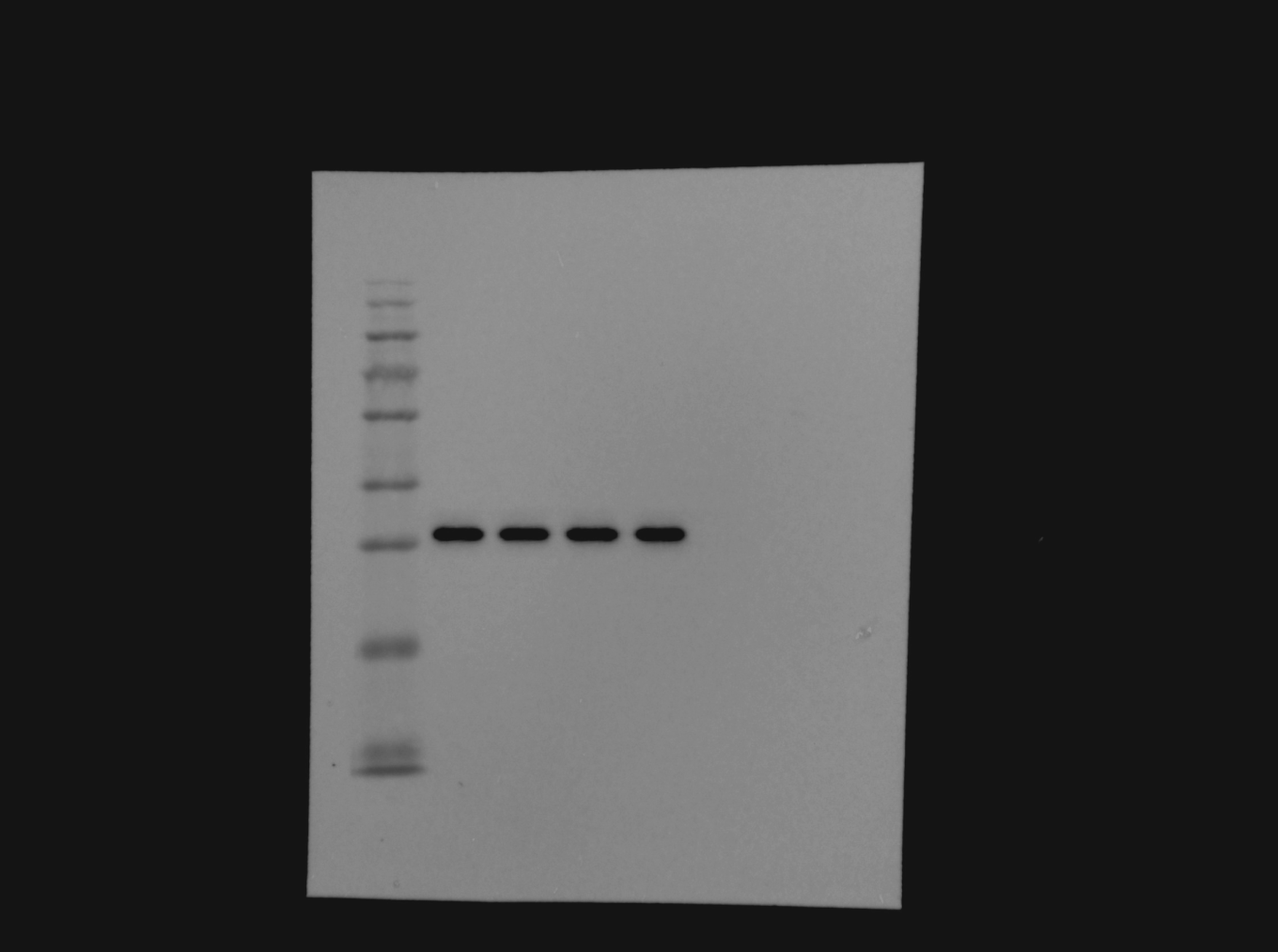
 S1D-6

Figure S4D:


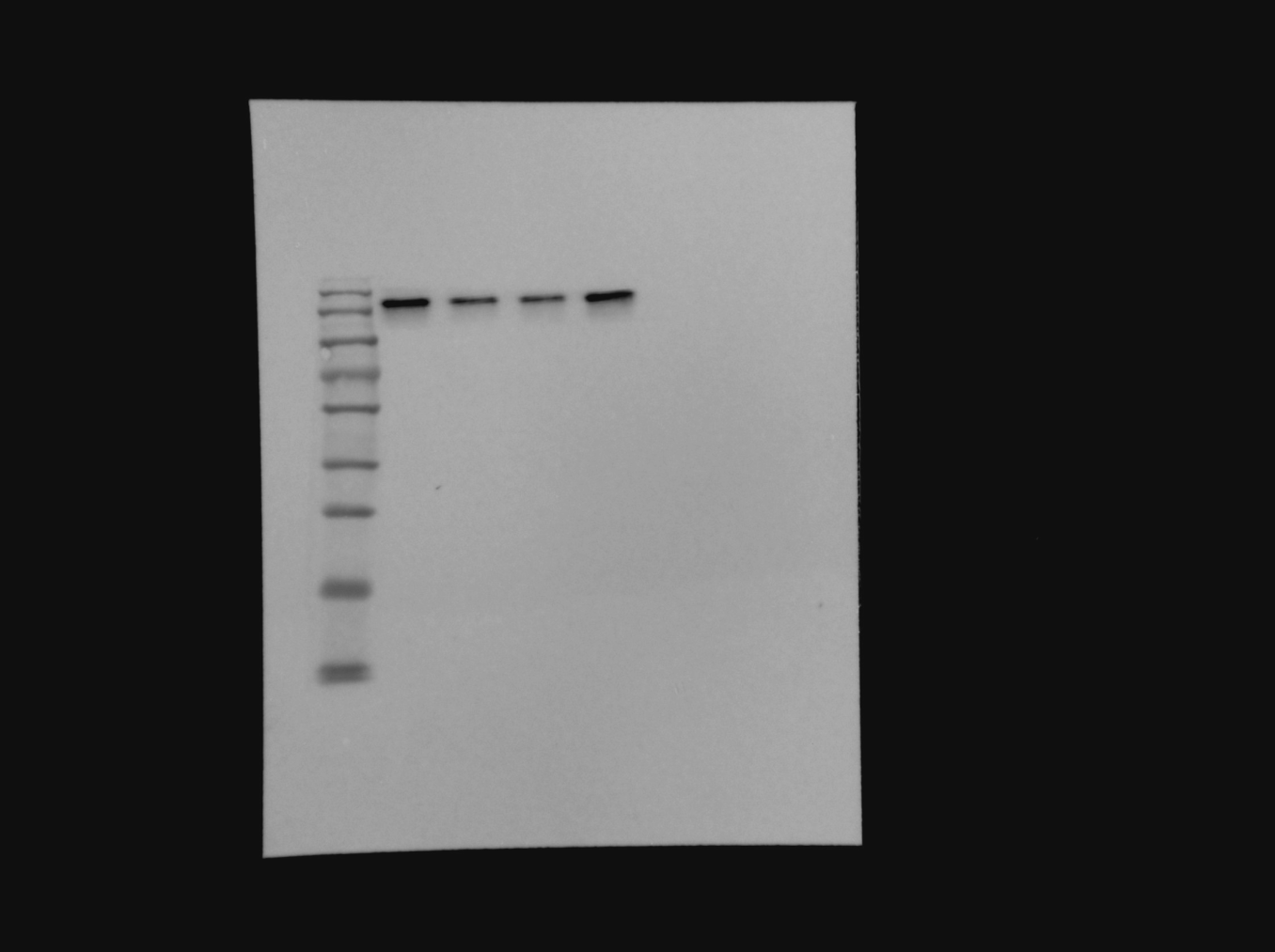
 S4D-1


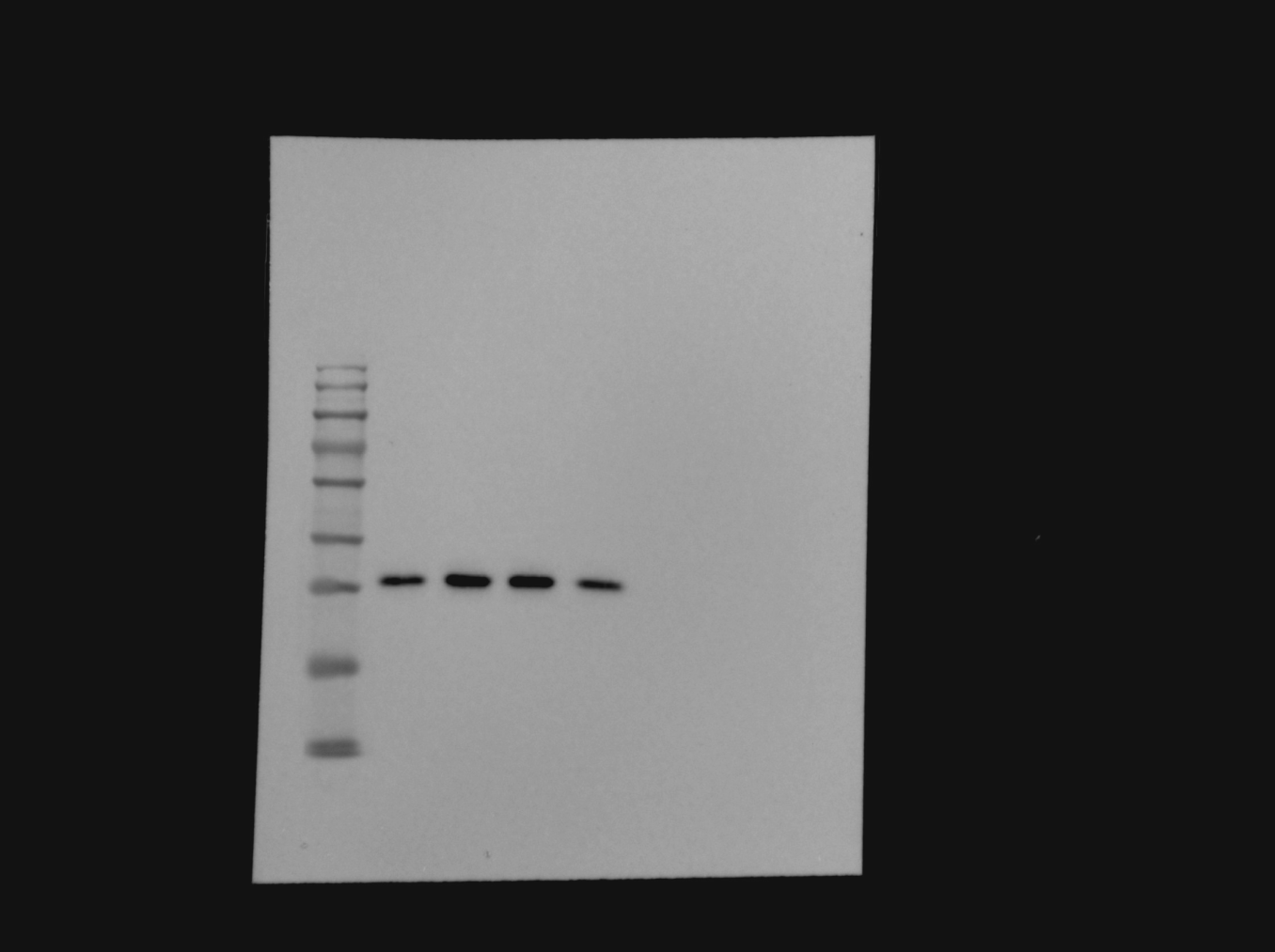
 S4D-2


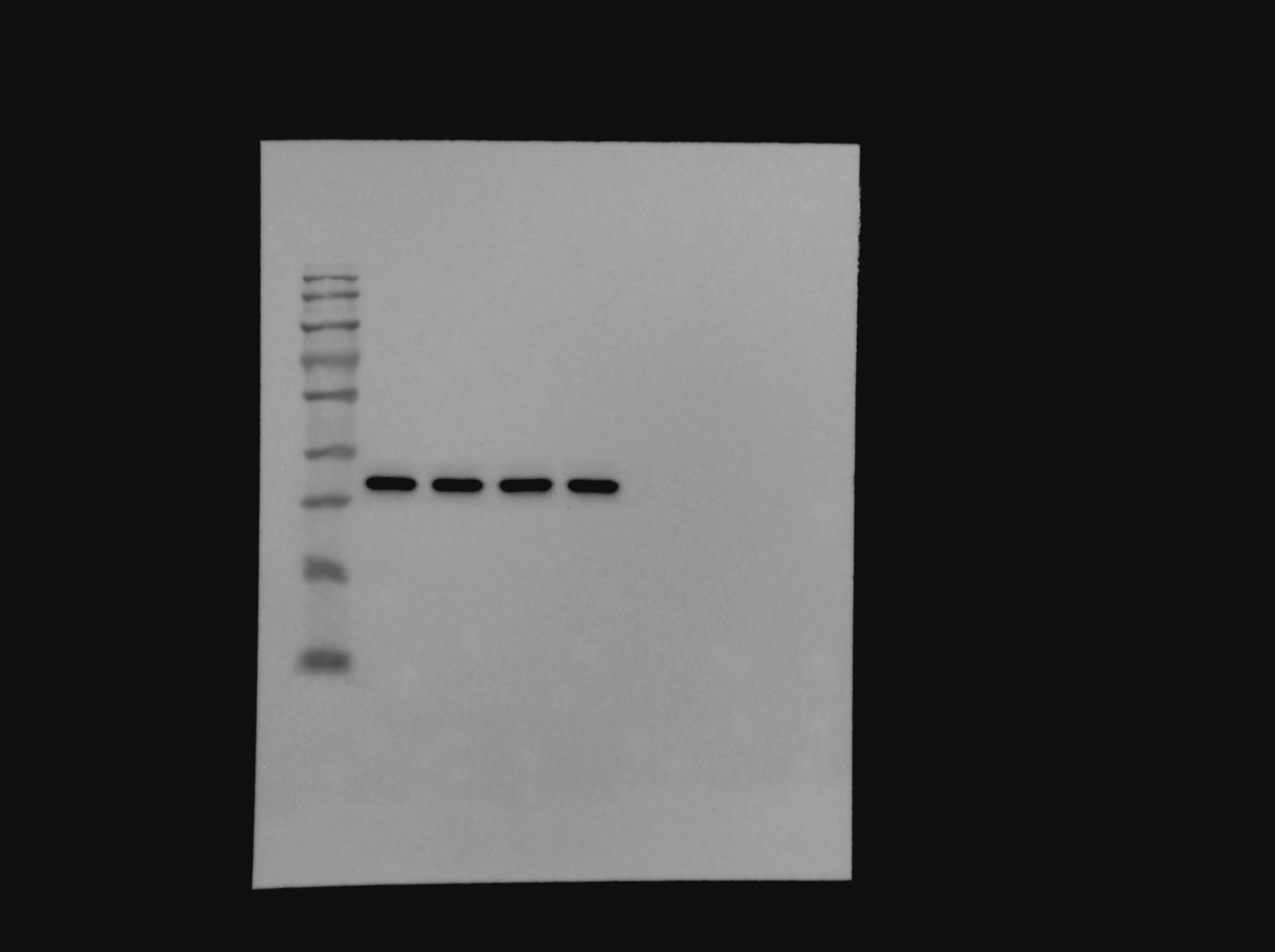
 S4D-3
